# Supplementary material for: Landscape of the Plasmodium Interactome Reveals Both Conserved and Species-Specific Functionality
Source: Cell Rep. 2019 Aug 6;28(6):1635–1647.e5. doi: 10.1016/j.celrep.2019.07.019 (PMC6693557; doi:10.1016/j.celrep.2019.07.019)
Supplement: Document S1. Figures S1 and S2 [file mmc1.pdf]

Cell Reports, Volume *28*

Supplemental Information

Landscape of the *Plasmodium* Interactome Reveals Both Conserved and Species-Specific Functionality

Charles Hillier, Mercedes Pardo, Lu Yu, Ellen Bushell, Theo Sanderson, Tom Metcalf, Colin Herd, Burcu Anar, Julian C. Rayner, Oliver Billker, and Jyoti S. Choudhary

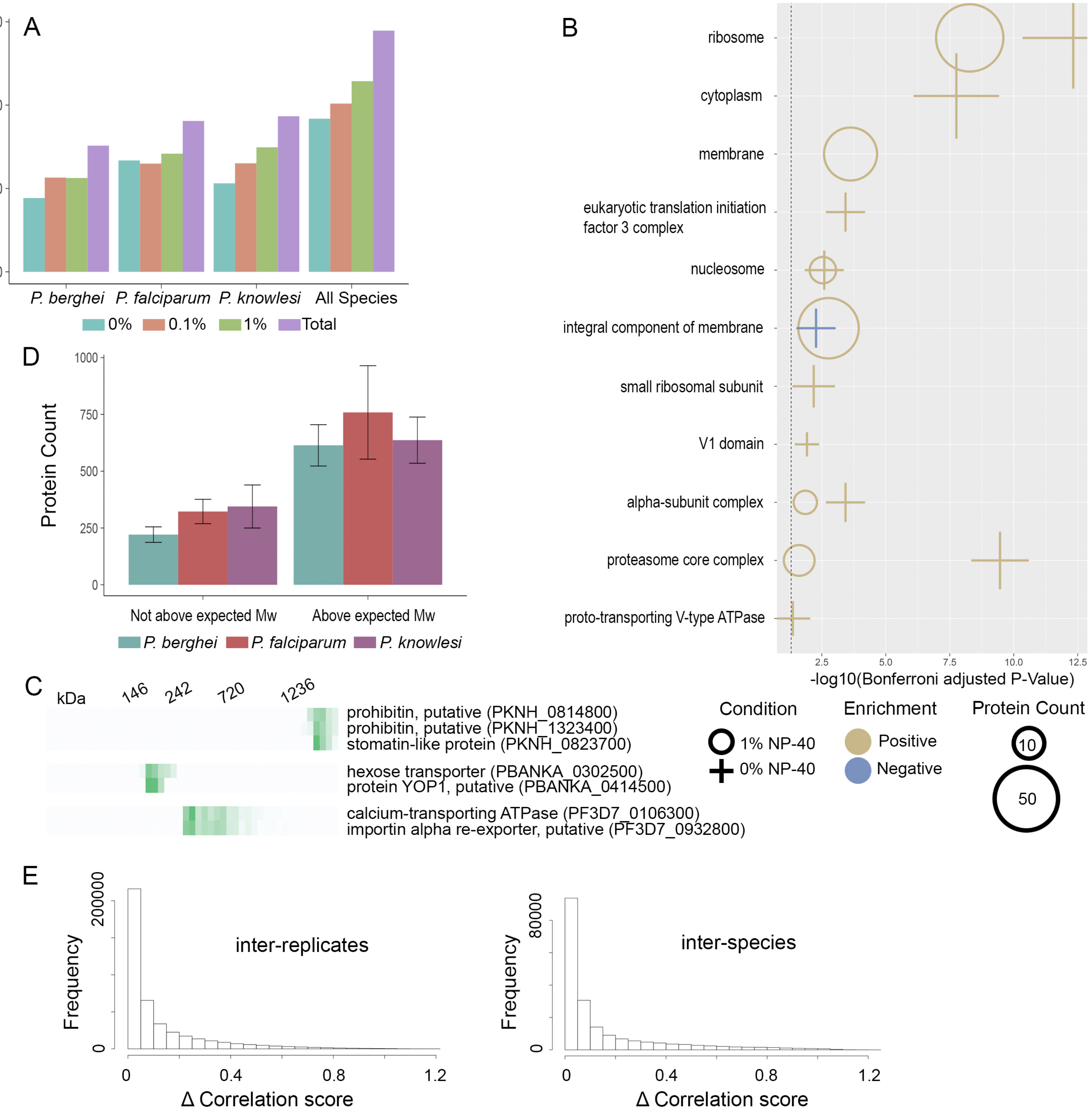

Fig. S1. Related to Figure 1. Description of the BN-PAGE fractionation data. (A) Number of proteins detected for each species and experimental condition. Bars are coloured by percentage of detergent in the lysis buffer. Protein counts for replicates are shown as union. (B) Comparison of positive and negative GO_CC term enrichments between *P. knowlesi* fractionation experiments in 0% and 1% NP-40 lysis conditions. Significance was assessed using Fisher's exact test with Bonferroni correction. The top 10 GO terms for each condition and enrichment with $p < 0.05$ are shown. The dashed line represents a p value of 0.05. (C) Migration profiles of membrane protein complexes only identified from lysates containing NP-40. (D) Number of detected proteins running above their expected BN-PAGE migration distance. Proteins with a predicted Mw at 30% higher than their literature Mw based on a regression model of known markers are considered to be running above their expected Mw. Error bars represent one standard deviation. (E) Histograms of absolute pairwise distances between co-migration scores of common pairs from replicate lysis conditions (*P. falciparum* 0.1% NP-40) and between species datasets (*P. berghei* 1% NP-40 and *P. falciparum* 0% NP-40).

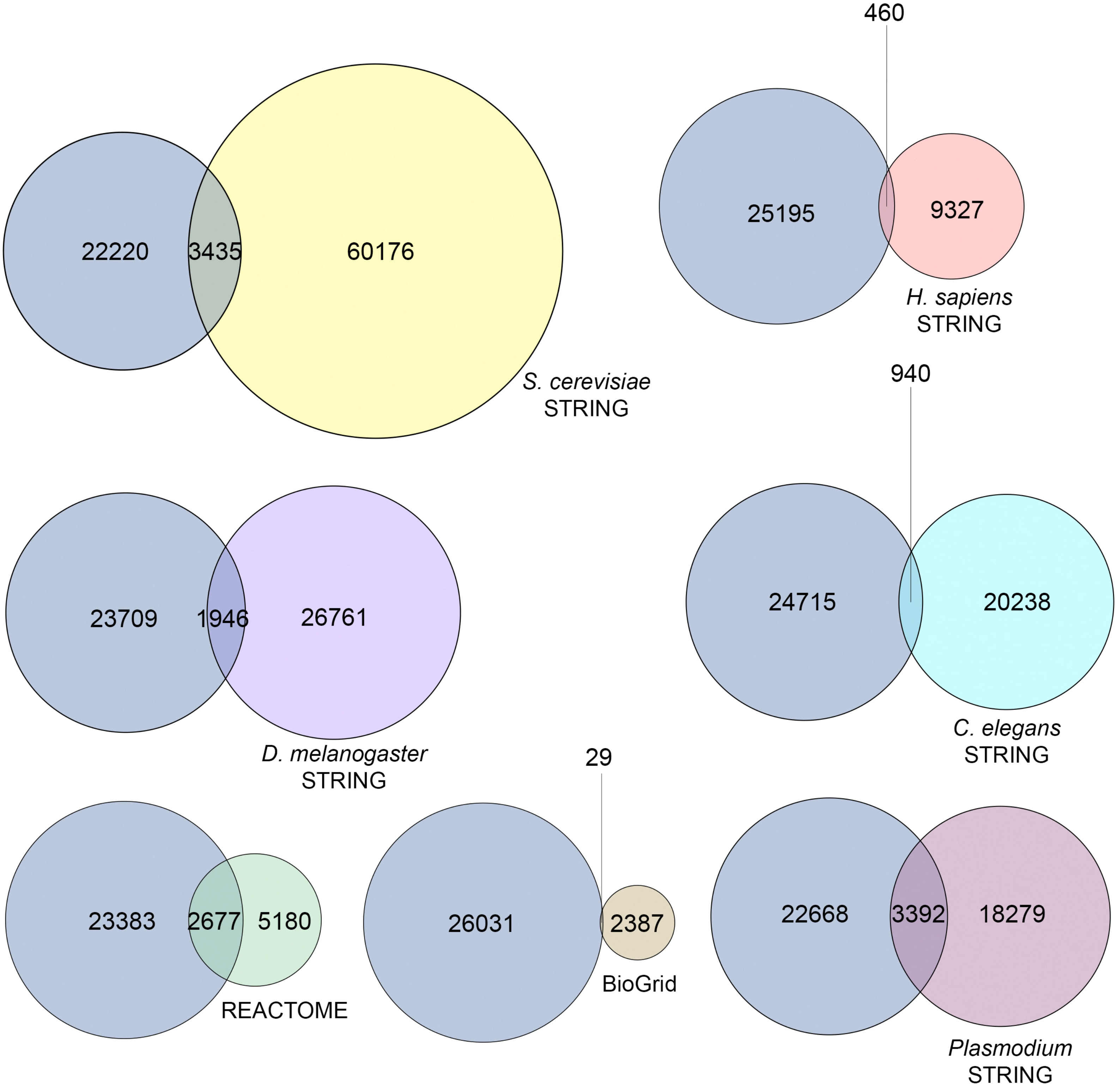

Fig. S2. Related to Figure 2. Evaluation of the high-confidence protein interaction network. Overlap between protein interactions from the random forest classifier output (grey) and PPIs annotated in public databases.
