# Supplementary material for: Landscape of the Plasmodium Interactome Reveals Both Conserved and Species-Specific Functionality
Source: Cell Rep. 2019 Aug 6;28(6):1635–1647.e5. doi: 10.1016/j.celrep.2019.07.019 (PMC6693557; doi:10.1016/j.celrep.2019.07.019)
Supplement: Document S2. Article plus Supplemental Information [file mmc11.pdf]

Resource

Cell Reports

Landscape of the *Plasmodium* Interactome Reveals Both Conserved and Species-Specific Functionality

Graphical Abstract

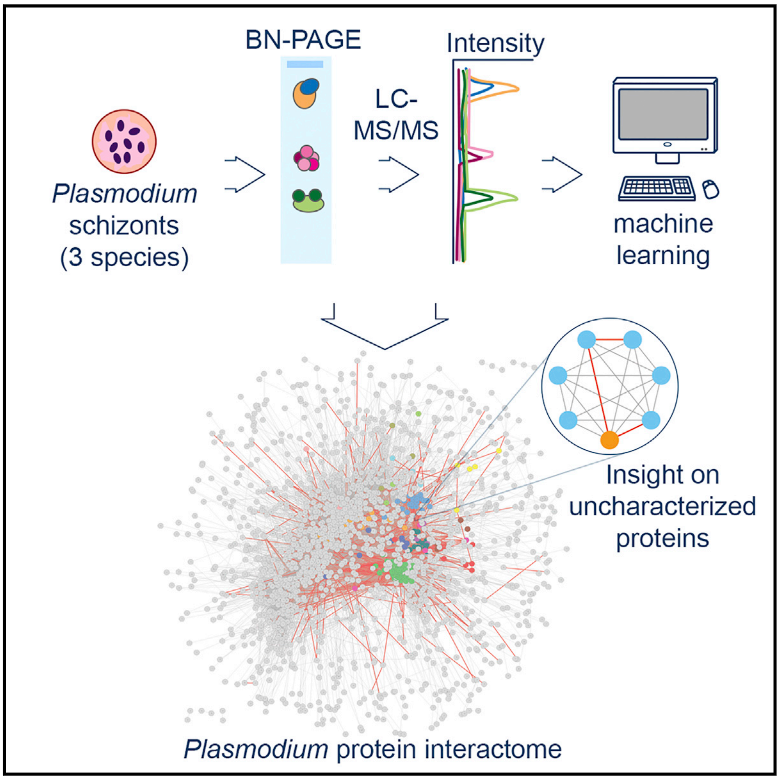

Authors

Charles Hillier, Mercedes Pardo, Lu Yu, ..., Julian C. Rayner, Oliver Billker, Jyoti S. Choudhary

Correspondence

mercedes.pardocalvo@icr.ac.uk (M.P.),
oliver.billker@umu.se (O.B.),
jyoti.choudhary@icr.ac.uk (J.S.C.)

In Brief

More than one-third of *Plasmodium* genes are uncharacterized functionally. Hillier et al. combine biochemical fractionation, protein correlation profiling, and machine learning to generate a protein interaction network for *Plasmodium*. This global cellular organization map sheds light on the function of known and uncharacterized proteins and highlights conserved and species-specific functionality.

Highlights

- Systems-level protein interaction network of *Plasmodium* schizonts
- The interactome facilitates inference of function for uncharacterized proteins
- Mechanistic insight into AP2-domain transcription factors' function
- Comparison of several species reveals conserved and species-specific functionality

Hillier et al., 2019, Cell Reports *28*, 1635–1647
August 6, 2019 © 2019 The Author(s).
https://doi.org/10.1016/j.celrep.2019.07.019

CellPress

Cell Reports
Resource

OPEN ACCESS
CellPress

Landscape of the *Plasmodium* Interactome Reveals Both Conserved and Species-Specific Functionality

Charles Hillier,[1,6] Mercedes Pardo,[2,6,7,*] Lu Yu,[2] Ellen Bushell,[3] Theo Sanderson,[4,5] Tom Metcalf,[4] Colin Herd,[4] Burcu Anar,[4] Julian C. Rayner,[4] Oliver Billker,[3,*] and Jyoti S. Choudhary[2,*]
[1]Developmental Biology Unit, European Molecular Biology Laboratory, 69117 Heidelberg, Germany
[2]Functional Proteomics, The Institute of Cancer Research, London SW7 3RP, UK
[3]Department of Molecular Biology, The Laboratory for Molecular Infection Medicine Sweden, Umeå University, 901 87 Umeå, Sweden
[4]Wellcome Sanger Institute, Wellcome Genome Campus, Cambridge CB10 1SA, UK
[5]Present address: Francis Crick Institute, London NW1 1ST, UK
[6]These authors contributed equally
[7]Lead Contact
*Correspondence: mercedes.pardocalvo@icr.ac.uk (M.P.), oliver.billker@umu.se (O.B.), jyoti.choudhary@icr.ac.uk (J.S.C.)
https://doi.org/10.1016/j.celrep.2019.07.019

SUMMARY

Malaria represents a major global health issue, and the identification of new intervention targets remains an urgent priority. This search is hampered by more than one-third of the genes of malaria-causing *Plasmodium* parasites being uncharacterized. We report a large-scale protein interaction network in *Plasmodium* schizonts, generated by combining blue native-polyacrylamide electrophoresis with quantitative mass spectrometry and machine learning. This integrative approach, spanning 3 species, identifies >20,000 putative protein interactions, organized into 600 protein clusters. We validate selected interactions, assigning functions in chromatin regulation to previously unannotated proteins and suggesting a role for an EELM2 domain-containing protein and a putative microrchidia protein as mechanistic links between AP2-domain transcription factors and epigenetic regulation. Our interactome represents a high-confidence map of the native organization of core cellular processes in *Plasmodium* parasites. The network reveals putative functions for uncharacterized proteins, provides mechanistic and structural insight, and uncovers potential alternative therapeutic targets.

INTRODUCTION

Plasmodium parasites caused 216 million new cases of malaria in 2016 and nearly 500,000 deaths (World Health Organization, 2017). The lack of an effective vaccine and spread of resistance to frontline antimalarial treatments make the search for new intervention targets a repeated and important research priority (Cowman et al., 2016). Target identification requires a better understanding of parasite biology and the underlying molecular mechanisms of parasite development and pathogenesis. Despite being the object of intense research efforts, more than one-third of *Plasmodium* genes still lack functional annotation, in large part because they lack direct orthologs outside closely related parasite species. While systematic genetic screening efforts are filling some of this gap (Bushell et al., 2017; Zhang et al., 2018), genetic data alone are often not enough to provide details of the function of the encoded proteins.

Physical associations between proteins, either transient or stable, in the form of protein complexes, are central to cellular processes. Elucidating protein-protein interactions (PPIs) can therefore assist in placing proteins in cellular pathways or biological processes and ascribing function to poorly characterized proteins. Attempts to elucidate PPI networks in *Plasmodium* parasites have so far relied primarily on *in silico* analyses of functional association information, including gene expression and homology (Ramaprasad et al., 2012). Large-scale experimental evidence has been limited to yeast two-hybrid studies, covering only ~22% of the *P. falciparum* proteome and relying on the expression of peptides in a non-native context (LaCount et al., 2005). In other organisms, systematic pull-down studies have been effective in elucidating PPI networks (Gavin et al., 2002, 2006; Hein et al., 2015; Huttlin et al., 2015, 2017), but lack of proteome-scale panels of antibodies and limitations in genetic manipulation that restrict high-throughput protein tagging in *Plasmodium* make this costly and time-consuming approach unfeasible, particularly to apply in several species. High-throughput chromatographic fractionation combined with quantitative mass spectrometry has emerged recently as an alternative strategy to elucidate protein complexes at the systems level and has been applied in organisms ranging from bacteria to humans (Crozier et al., 2017; Havugimana et al., 2012; Kastritis et al., 2017; Kirkwood et al., 2013; Kristensen et al., 2012; Wan et al., 2015). This approach provides a global analysis of the interactome and does not require any genetic manipulation or affinity reagents. Blue native (BN)-PAGE, which separates protein complexes in native conformation based on Coomassie brilliant blue binding, has a higher resolution than gel filtration or sucrose density centrifugation and has proven to be particularly useful in resolving membrane protein complexes (Bode et al., 2016; Heide et al., 2012; Schägger et al., 1994; Schägger and von Jagow, 1991), which are often underrepresented in high-throughput datasets. Here, we used BN-PAGE fractionation coupled to

This is an open access article under the CC BY license (http://creativecommons.org/licenses/by/4.0/).

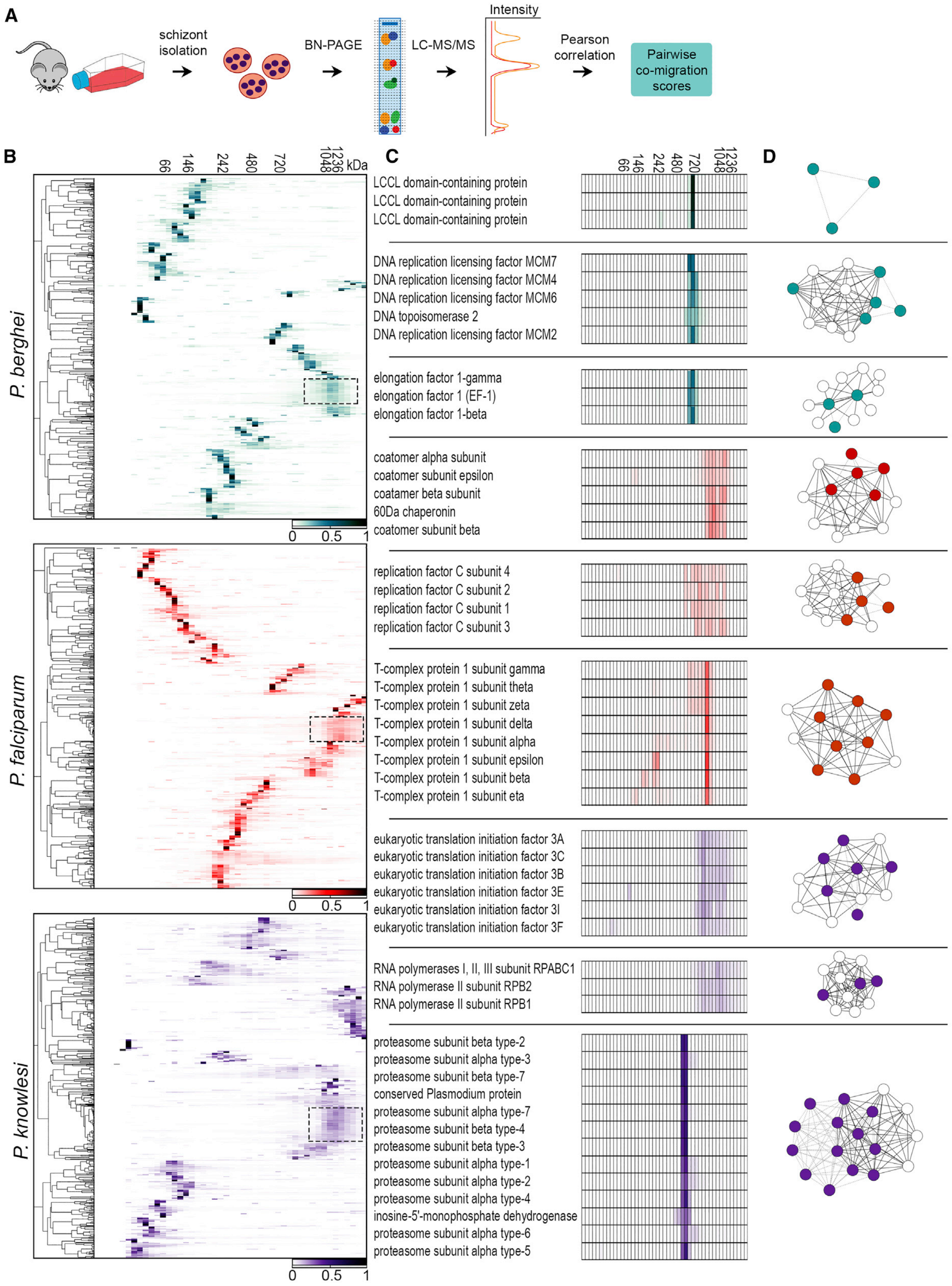

(legend on next page)

quantitative mass spectrometry-based correlation profiling in combination with supervised machine learning (global BN protein correlation mass spectrometry [GBC-MS]) to build a high-confidence PPI network for *Plasmodium*.

RESULTS

Plasmodium Complexome Profiling Using BN-PAGE Coupled to Tandem MS

To produce a comprehensive and accurate protein interaction network in *Plasmodium*, we applied a strategy based on high-throughput biochemical fractionation using BN-PAGE coupled to quantitative tandem MS to three different species, namely *P. falciparum*, *P. knowlesi*, and *P. berghei. P. falciparum* is the cause of most human malaria mortality and is the dominant malaria parasite in Africa. *P. knowlesi* is a zoonotic pathogen that causes thousands of cases of malaria every year but also serves as a valuable *in vitro* model for *P. vivax*, the most common cause of malaria outside Africa but which cannot be cultured *in vitro*, making it inaccessible for proteomics studies. *P. berghei* is a widely used rodent model for malaria and has the twin advantages of being an *in vivo* model and one that is highly amenable to genetic manipulation. Because the clinical symptoms of malaria are caused by the asexual erythrocytic parasites, we focused on the schizont stage. To achieve broad cellular coverage, we used several detergent concentrations (0, 0.1, and 1% Nonidet P-40) during cell lysis, aiming to balance solubilization in native conditions with protein recovery. Although the use of detergent may disrupt some protein interactions, the low concentrations used here are commonly applied in interaction studies and should help enhance the coverage of membrane proteins. We performed proteome fractionation of schizont lysates by BN-PAGE in duplicate for each experimental condition (3 *Plasmodium* species with 3 detergent concentrations), collected 48 fractions per experiment, and analyzed each fraction by quantitative liquid chromatography-tandem MS (LC-MS/MS) (Figure 1A). In total, we gathered 18 high-throughput fractionation datasets comprising 864 fractions, yielding a collection of 3,085,858 MS/MS spectra that were attributed to *Plasmodium* peptides (Table S1).

By mapping the gene identifications for each species to *P. falciparum*, we detected a total of 2,894 unique proteins (Table S1), representing >50% of the proteins encoded in the *Plasmodium* genome (Figure S1A). Given that not all genes are expressed in any given parasite stage, this is likely to represent the majority of schizont proteins. Our dataset overlapped with 74% of proteins identified in the deepest malaria proteome to date, containing 1,673 proteins identified by >1 peptide, also from schizonts (Treeck et al., 2011), and identified 537 additional proteins. We detected a greater number of proteins when using higher detergent concentrations, as expected (Figure S1A). To compare the properties of identified proteins between our experimental datasets, we performed Gene Ontology (GO) enrichment analysis (Table S2). The GO term “integral component of membrane” was under-represented in datasets without detergent (Fisher’s exact test with Bonferroni correction, $p < 0.05$) but not in the presence of detergent (Figure S1B). In keeping with the increased solubilization of membrane proteins with increasing detergent concentrations, we detected some membrane complexes only after detergent extraction (Figure S1C). This illustrates the enhanced capability of BN-PAGE to resolve both soluble and membrane protein complexes when compared with other fractionation systems (Havugimana et al., 2012; Kirkwood et al., 2013). More than 60% of proteins ran significantly above their predicted molecular weight, indicating that the majority of proteins migrated as part of the higher-order assemblies (Figure S1D).

Next, we quantified protein abundances using extracted MS1-based intensities across the 48 fractions in each experimental condition to derive protein migration profiles for each of the proteins identified (Figure 1A). To assess the faithfulness of the biochemical fractionation, we performed hierarchical clustering of protein migration profiles (Figure 1B). The clustered profiles of all three *Plasmodium* species showed remarkable similarity, suggesting a conserved core network of functional units. Subunits of well-characterized conserved housekeeping complexes with a wide range of sizes, such as proteasome, ribosome, nucleosome, chaperonin containing TCP1 (CCT) complex, elongation factor 1, and minichromosome maintenance protein (MCM) complex, showed highly correlated co-migration profiles that clustered together (Figures 1C and 1D). We also observed tightly overlapping profiles between subunits of malaria-specific complexes such as the *Limulus* clotting factor C, Coch-5b2, and Lgl1 (LCCL)-lectin adhesive-like protein (LAP) core complex, which is involved in parasite development and infectivity (Saeed et al., 2010; Simon et al., 2009). This complex was identified only in *P. berghei* fractionations, despite being conserved across *Plasmodium* (Pradel et al., 2004). This likely reflects the presence of a small number of LCCL-expressing gametocytes in the *P. berghei* schizont preparation, highlighting that our strategy could be useful in resolving life cycle stage-specific protein complexes. For each pair of proteins, we generated a co-migration

Figure 1. Identification of *Plasmodium* Protein Complexes

(A) Schematic overview of the blue native polyacrylamide gel electrophoresis (BN-PAGE) strategy used to derive a protein interaction network. *Plasmodium* schizont lysates were subjected to BN-PAGE and migration profiles generated for each protein using MS1 peak intensities measured by quantitative LC-MS/MS. Two biological replicates were carried out for each condition. Profiles were correlated to generate pairwise co-migration scores, which were then used to build a network.

(B) Representative heatmaps derived from hierarchical clustering of protein migration profiles for *P. berghei* (green), *P. falciparum* (red), and *P. knowlesi* (purple). Dendrograms are shown at left. Clustering was performed with Pearson correlation, complete linkage, and pre-processing with K-means. Protein markers (molecular weight) are shown at the top. Scale bars represent normalized intensity. The dotted box indicates the ribosome.

(C) Migration profiles of select examples of known complexes. Protein descriptions from PlasmoDB are shown.

(D) Network graphs of protein complexes shown in (C). Colored nodes represent co-migrating proteins identified here. White nodes represent other known interactors annotated in STRING. Solid edges are found in StringDB. Dashed edges are derived from co-migration only.

See also Tables S1 and S2 and Figure S1.

score by correlating their migration profiles, and this score was later used for assembling co-migration networks through machine learning. To assess reproducibility between experimental profiles, we compared co-migration scores for pairs of proteins by plotting the distribution of absolute differences between common protein pairs. More than 50% of protein pairs displayed co-migration score differences smaller than 0.05 between biological replicates, and a similar degree of reproducibility was observed between *Plasmodium* species datasets (Figure S1E), allowing data integration in subsequent analyses. Our data illustrate the validity of the BN-PAGE fractionation and protein correlation profiling strategy and overall procedure in recapitulating bona fide protein complexes.

Generation of a *Plasmodium* Protein Interaction Network

To generate a high-confidence PPI network, we used a random forest machine learning approach (Havugimana et al., 2012) that integrated the co-migration scores derived from the biochemical fractionation with additional information supporting physical association (Figure 2A). The rationale behind this is that physically interacting proteins carry out related biological functions, are co-expressed, and often have similar evolutionary conservation, and these features can be used to enhance the confidence when deriving interactions from the experimental migration profiles. Only protein pairs with strong biochemical evidence from the fractionation datasets (correlation score of at least 0.4) were used, and additional supporting features were applied to this subset. We included 2 additional measures derived from biochemical fractionation data reflecting reproducibility, namely the number of fractionation experiments in which the protein pair had a co-migration score of at least 0.4 and the number of fractionation experiments in which the maximal peak in the migration profile overlapped for each protein pair. Other evidence supporting functional association included gene co-expression (Bozdech et al., 2003; Hu et al., 2010; Modrzynska et al., 2017), interacting domains (Raghavachari et al., 2008; Yellaboina et al., 2011), co-evolution (Juan et al., 2008; Ochoa et al., 2015), and phenotypic data (Bushell et al., 2017) (Table S3). The machine learning classifier was trained and tested with a gold standard set comprising only experimentally determined *Plasmodium* interactions annotated in the STRING database (Szklarczyk et al., 2017) (see Method Details for details). Assessment of the relative contribution of each feature to the prediction of PPIs (as measured by the Gini score) confirmed that the biochemical evidence collectively had the biggest impact on the classification (Table S3), reflecting the superior power of co-migration compared to other functional association information in predicting interactions. To measure the overall performance of the classifier, we performed receiver operating characteristic analysis of the high-confidence PPIs against the gold standard test set. This revealed a significant improvement in recalling true interactions for the classifier compared to the co-fractionation data alone (Figure 2C). After applying a random forest (RF) score threshold of 0.9, based on the maximal overlap of protein clusters with annotated protein complexes (see below), the machine learning analysis yielded a network of 1,761 *Plasmodium* proteins and 26,060 interactions (Figure 2B; Tables S4 and S5). More stringent filtering such as increasing the RF score threshold or introducing additional criteria (e.g., requiring a co-migration score of at least 0.4 in at least 2 experiments) did not result in a significant increase in true-positive recall but led to the loss of interactions that were subsequently validated experimentally (see below).

We also tested performance against other interaction datasets not used in the training (Figure S2). The PPI network recapitulated 16% of *Plasmodium* experimentally determined interactions annotated in STRING. Only 1% of the interactions for *P. falciparum* in BioGrid, comprising mainly yeast two-hybrid data (LaCount et al., 2005), were captured, indicating a significant divergence between our native PPI network and that inferred from the heterologous expression of peptides. It is worth noting that this yeast two-hybrid interactome, the sole experimental systems level *Plasmodium* interactome published to date, has low coverage and is based on the expression of small-sized adenine + thymine (AT)-rich *Plasmodium* protein fragments, and thus the disagreement in the two sets is hardly surprising. Our network recapitulated 33% of the interactions derived from REACTOME, and replicated interactions previously described in other eukaryotic organisms. We found 3,632 PPIs that have previously been reported for orthologs in yeast, worm, fly, or human (as annotated in STRING, experimental interactions only, minimum of 0.4 evidence score), with one-third of these reported in yeast but not in metazoans. Fewer than 200 PPIs have been reported in metazoans but not in yeast. For instance, we detected an interaction between *Plasmodium* Rab guanosine diphosphate (GDP) dissociation inhibitor (GDI) and a farnesyltransferase; this interaction is annotated for *Plasmodium* in STRING based on the experimental evidence of putative orthologs interacting in *Saccharomyces cerevisiae*. Rab GDIs bind to prenylated Rab proteins that regulate vesicular trafficking and delimit membrane structures, delivering them to and retrieving them from their membrane-bound compartment (Pfeffer et al., 1995). Our data demonstrate that this interaction does indeed occur in *Plasmodium*. Overall, the PPIs described here represent a substantial increase in the number of apicomplexan interactions reported to date.

Inferring Putative Protein Function for *Plasmodium* Proteins from the Interaction Network

To delineate distinct functional units from the network, we performed cluster analysis using the ClusterONE algorithm (Nepusz et al., 2012). The resulting 593 clusters, comprising 1,259 unique proteins, represent putative protein complexes ranging in size between 2 and 74 nodes (Figure 2D; Table S6). The clusters recapitulated 89 complexes previously reported in CORUM (Tables S6 and S7). The clusters are involved in a wide variety of biological processes, and 59 of them showed enrichment of GO biological function terms (Table S7). Hence, our PPI network is able to provide high-resolution information on malaria protein complexes and/or functional protein assemblies.

We identified several clusters representing well-characterized malaria-specific protein complexes (Figure 3A), including the *Plasmodium* translocon of exported proteins (PTEX), responsible for protein export from the parasitophorous vacuole (de Koning-Ward et al., 2009), the adaptor protein 1 (AP-1) complex, which

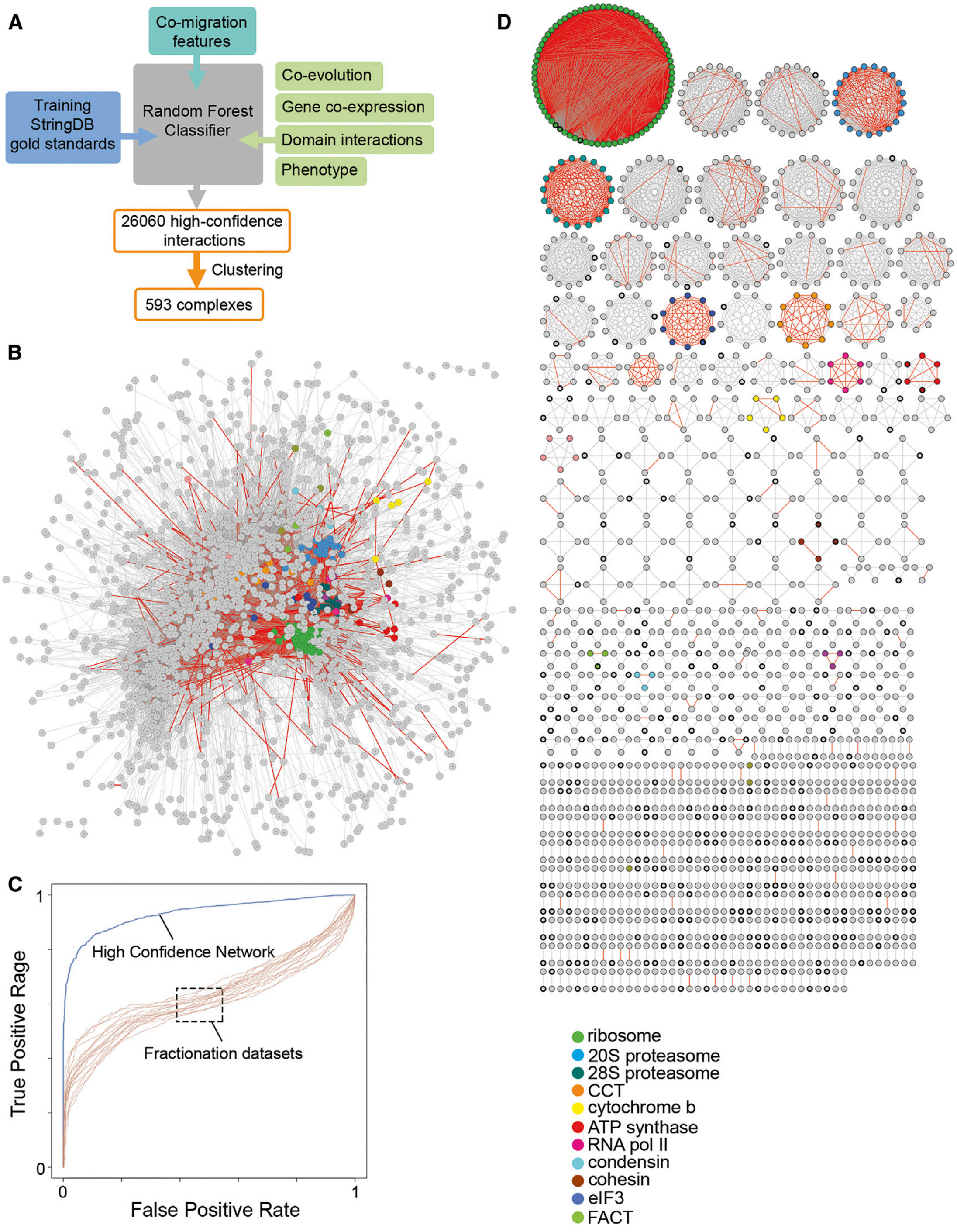

Figure 2. Generation of a High-Confidence *Plasmodium* Protein Interaction Network

(A) Schematic of the machine learning pipeline applied to the BN-PAGE fractionation data. Pairwise co-migration scores were supported with functional association information using a random forest classifier trained with a gold standard set derived from STRING.

(B) Global *Plasmodium* PPI network derived through BN-PAGE correlation profiling (GBC-MS) and machine learning.

(C) Receiver operating characteristic analysis of BN-PAGE fractionation experiments (brown, mean area under the curve [AUC] = 0.63, SD = 0.023) and the random forest classifier output (blue, AUC = 0.94). Performance was assessed against a gold standard set derived from STRING.

(D) Protein clusters representing putative protein complexes. Conserved *Plasmodium* proteins of unknown function are shown with a thick border.

For (C) and (D), the examples of well-known complexes are colored. The red edges represent interactions annotated in STRING.

See also Tables S3, S4, S5, S6, and S7 and Figure S2.

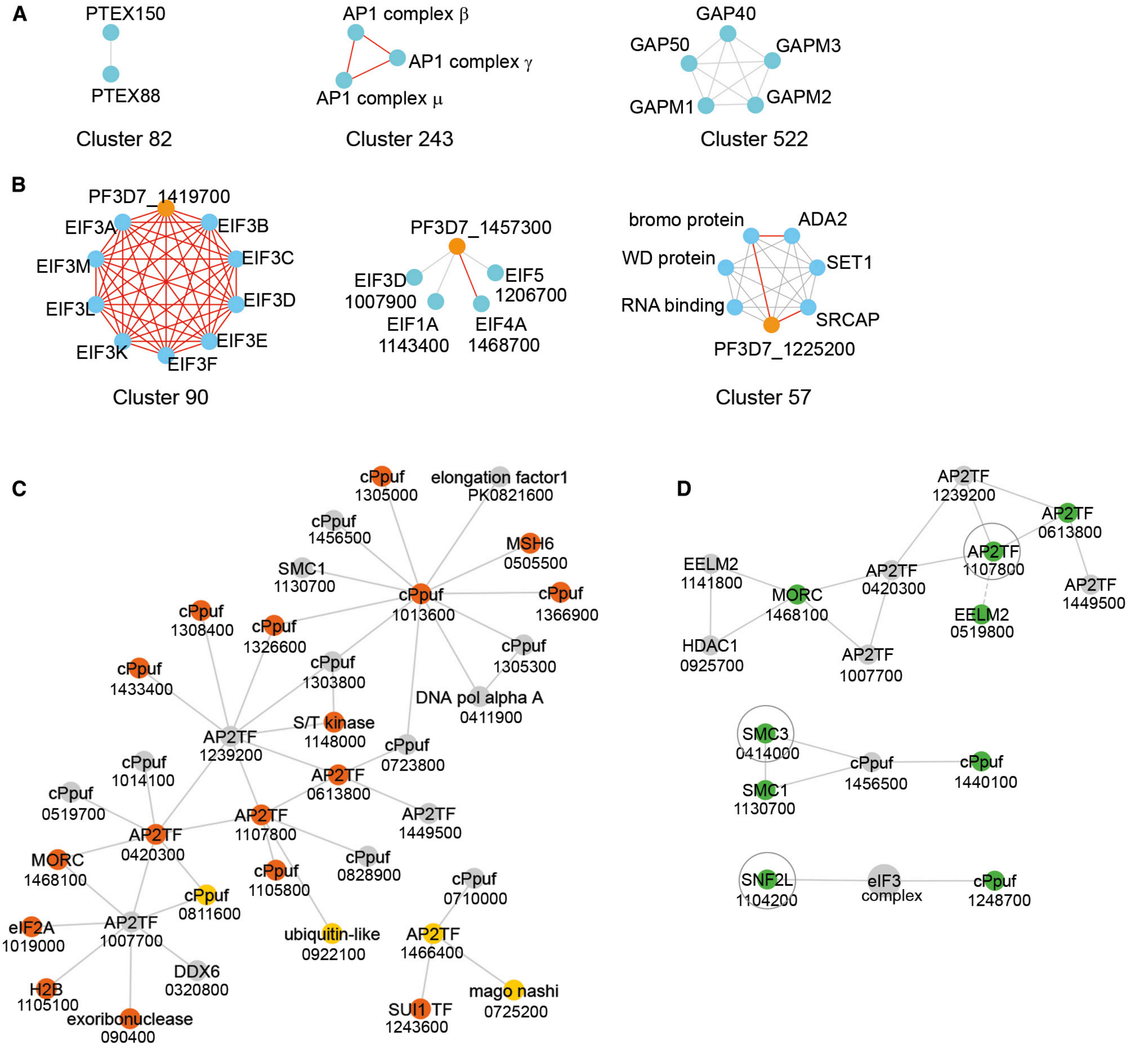

Figure 3. Protein Complex Membership for Predicting the Function of Malaria Uncharacterized Proteins

(A) Examples of clusters representing known malaria-specific protein complexes PTEX (de Koning-Ward et al., 2009), AP-1 (Kaderi Kibria et al., 2015), and glideosome (Frénal et al., 2017) found in this study.

(B) Examples of clusters containing conserved *Plasmodium* proteins of unknown function (in orange). Red edges represent interactions annotated in STRING.

(C) The ApiAP2 transcription factor interaction network. Relevant first-order interactions involving ApiAP2 transcription factors were extracted from the PPI network. Nodes labeled cPpuf are conserved *Plasmodium* proteins of unknown function. Orange nodes are essential proteins, and yellow nodes represent proteins whose mutation results in slow growth.

(D) Validation of interactions by affinity purification-mass spectrometry from tagged *P. berghei* lines. Represented are subsections of the PPI network. The baits are surrounded by a circle. The proteins identified specifically in the immunoprecipitate of each bait are in green. The data are from two independent biological repeats. The dotted line represents an indirect link in the network.

The numeric part of *P. falciparum* gene names is shown in (B)–(D).

See also Table S8.

has a role in protein trafficking to rhoptry organelles (Kaderi Kibria et al., 2015), and the glideosome, a structure involved in merozoite entry into the host erythrocyte during invasion (Frénal et al., 2017). All of these are examples of membrane protein complexes, which further highlight the utility of our BN-PAGE approach in resolving these less-soluble assemblies that would be missed by alternative chromatographic fractionation approaches. Previous interactome studies based on

Table 1. Function Prediction for Selected Conserved *P. falciparum* Proteins of Unknown Function

Protein ID	Predicted Function	Supporting Evidence
PF3D7_1454200	transcription	ClusterGO: transcription
PF3D7_1142800	ATP synthesis coupled proton transport	ClusterGO: ATP synthesis coupled proton transport
PF3D7_1024300	ATP synthesis coupled proton transport	ClusterGO: ATP synthesis coupled proton transport
PF3D7_1225200	epigenetic regulation	contains DNA-binding domains
		ClusterGO: DNA-templated
PF3D7_0210900	translation	slow growth
		ClusterCORUM: ribosome
PF3D7_1419700	eIF3 complex subunit	annotated as eIF3H in STRING
		ClusterCORUM: eIF3 complex
PF3D7_1314300	chromatin biology	macro domain-like
		ClusterCORUM: SMC1-SMC3 complex
PF3D7_1456500	cohesin complex	STAG domain
		ClusterCORUM: SMC1-SMC3 complex
PF3D7_1024300	ATP synthase subunit or binding protein	ClusterCORUM: F1F0-ATP synthase (EC 3.6.3.14), mitochondrial
PF3D7_0311400	phosphorylation of condensin subunits	ClusterCORUM: condensin complex
		ClusterGO: chromosome organization
PF3D7_1457300	scaffold for translation initiation factors	MA3 domain
PF3D7_1468100	AP2 TF driven transcription	interaction with several ApiAP2 transcription factors
PF3D7_1248700	chromatin remodeling	experimentally validated interaction with SNF2; link with RNA polymerase I
PF3D7_1440100	cohesin complex	experimentally validated interaction with cohesin complex
PF3D7_1012700	RNA polymerase II C-terminal domain (CTD) phosphatase	contains a CPDc/FCP1 homology domain (catalytic domain of CTD-like phosphatases)
		ClusterCORUM: RNA polymerase II core complex
		ClusterGO: DNA binding, DNA-directed RNA polymerase activity, ribonucleoside binding, transcription

The prediction was based on the protein being part of a cluster enriched in a particular GO term (ClusterGO) and/or CORUM complex (ClusterCORUM), supported in some cases from other evidence indicative of a physical association. Protein ID represents PlasmoDB accession number.

chromatographic fractionation have focused mainly on soluble complexes (Havugimana et al., 2012), and they could be complemented by BN-PAGE fractionation studies.

To explore malaria-specific interactions in a systematic manner, we focused on the conserved *Plasmodium* proteins of unknown function. The clustered network encompassed 215 of these proteins, distributed among 224 clusters (including 26 CORUM complexes), potentially representing malaria-specific subunits and/or complexes (Figure 2D; Table S6). A total of 15 conserved *Plasmodium* proteins belonged to clusters enriched for certain biological process GO terms and 11 belonged to CORUM clusters, and these data could inform their function through "guilt by association." The high-confidence PPI network can predict the function of poorly characterized or unannotated malaria proteins, associate them with specific aspects of parasite biology, or shed light on the mechanism of action (Table 1; Figure 3B). A conserved *Plasmodium* protein of unknown function, PF3D7_1419700, is part of a cluster with multiple subunits of translation initiation factor 3 (eIF3), and is annotated in STRING but not in PlasmoDB as a putative ortholog of human eIF3H. Our results demonstrate that PF3D7_1419700 interacts with the eIF3 translation initiation complex. Another conserved *Plasmodium* protein, PF3D7_1457300, interacts with different eIFs and contains overlapping multiple copies of the MA3 domain, a PPI domain present in eIF4G, suggesting that PF3D7_1457300 could be a scaffold for eIF4 and/or other translation initiation factors. A third conserved *Plasmodium* protein of unknown function, PF3D7_1225200, containing Myb-binding, SWIRM, and SANT domains, belongs to cluster 57, which also includes other DNA-binding proteins—transcriptional coactivator ADA2, a SET domain protein, a WD repeat protein, a protein containing a bromodomain (which binds acetylated histones [Owen et al., 2000]), and an Snf2h-related CBP activator; it also has a link with histone acetyltransferase GCN5. The domains in PF3D7_1225200 are also present in mammalian histone H2A deubiquitinase Mysm1, which interacts with histone acetyltransferase p/CAF (Zhu et al., 2007). The links between the WD repeat, bromodomain, and SET domain proteins are reminiscent of the interactions reported in mammalian cells (Bode et al., 2016; Dharmarajan et al., 2012; Dou et al., 2005; Revenko et al., 2010).

Sequence-specific regulators of transcription in Apicomplexa are characterized by functional *apetala2* (AP2) DNA-binding domains (Balaji et al., 2005). Mechanisms through which these ApiAP2 proteins regulate transcription have remained elusive, and it is therefore remarkable that we observed a complex of 3

Table 2. Validation of Protein Interactions by AP-MS on Tagged *P. berghei* Lines

P. berghei Accession	*P. falciparum* Accession	Description	Pb0939100 # Peps	Pb0939100 SP	Pb0716000 # Peps	Pb0716000 SP	Pb0942700 # Peps	Pb0942700 SP	Control # Peps (avg.)
PBANKA_0939100	PF3D7_1107800	transcription factor with AP2 domain(s), putative (ApiAP2)	59 | 48	1	0 | 2	0.01	0 | 5	0.09	1
PBANKA_1331400	PF3D7_1468100	conserved *Plasmodium* protein, unknown function	26 | 22	1	0 | 0	0	1 | 1	0	2
PBANKA_0112100	PF3D7_0613800	transcription factor with AP2 domain(s), putative (ApiAP2)	35 | 27	1	0 | 2	0	0 | 0	0	2
PBANKA_1234600	PF3D7_0519800	EELM2 domain-containing protein, putative	4 | 2	0.84	1 | 1	0	0 | 0	0	0
PBANKA_0716000	PF3D7_0414000	structural maintenance of chromosomes protein 3, putative	0 | 2	0	47 | 62	1	0 | 4	0.01	1
PBANKA_0917500	PF3D7_1130700	structural maintenance of chromosomes protein 1, putative	0 | 0	0	41 | 57	1	0 | 1	0	0
PBANKA_1304000	PF3D7_1440100	conserved *Plasmodium* protein, unknown function	0 | 0	0	11 | 16	1	0 | 0	0	0
PBANKA_0942700	PF3D7_1104200	chromatin remodeling protein, putative (SNF2L)	0 | 0	0	0 | 0	0	41 | 58	1	0
PBANKA_1461600	PF3D7_1248700	conserved *Plasmodium* protein, unknown function	0 | 0	0	0 | 0	0	46 | 38	1	0

Number of unique peptide sequences (# Peps) of specific interactors identified in duplicate AP-MS experiments on indicated bait protein (headers), SAINT probability score (SP) representing the specificity of the interaction, and average number of unique peptides in control experiments (n = 5). Gene identifications of orthologs in *P. falciparum* are shown. See also Table S8.

likely essential ApiAP2 proteins (Figure 3C; Table S6, cluster 454), 2 of which (PBANKA_1453700/PF3D7_1239200 and PBANKA_0939100/PF3D7_1107800) mark each of the 2 major gene expression clusters in the second half of the intraerythrocytic developmental cycle in both *P. berghei* and *P. falciparum* (Reid et al., 2018). Among the ApiAP2 TF second neighbors, we identified several DNA-binding proteins, including DNA polymerase I, DNA damage proteins, and putative epigenetic regulators (Table S6). Other interactors, such as a protein kinase, could be involved in the regulation of transcription factor activity and represent a potential therapeutic target.

Regulatory interactions such as those between enzyme and substrate may not be encompassed into clusters, yet are still found in the network. For example, we detected an interaction between merozoite surface protein 1 (MSP1) and putative protein arginine *N*-methyltransferase 5. MSP1 has previously been identified in a pull-down study of arginine-methylated proteins (Zeeshan et al., 2017); our data suggest that it could be a target of protein arginine methyltransferase 5 (PRMT5). Arginine methylation may regulate a broad array of *Plasmodium* physiological processes, and in fact, three other interactors of PRMT5 identified in our study were also previously found to be arginine methylated (Zeeshan et al., 2017). Overall, these examples illustrate the potential of our PPI network as a source of insight into individual complexes representing a wide array of parasite pathophysiology.

Validation of Protein Complexes from the *Plasmodium* Protein Interaction Network

To assess the precision of our study, we chose candidate interactions for validation based on the availability of reagents, similarity of GO terms with putative interacting proteins in the same cluster, and essential phenotype. We performed affinity purification coupled to MS experiments from *P. berghei* schizonts expressing endogenous hemagglutinin (HA)-tagged versions of selected candidates and validated several interactions from the network, in addition to discovering other interactions (Figures 3D and 3E).

To validate the proposed complex of ApiAP2 proteins, epitope-tagged PBANKA_0939100 was immunoprecipitated from the lysates of *P. berghei* schizonts, and MS identification of co-purifying proteins confirmed the interaction with PBANKA_0112100 (Tables 2 and S8). We additionally confirmed an association with two conserved *Plasmodium* proteins of unknown function. The first is a nuclear protein with an extended Egl-27 and MTA1 homology 2 (EELM2) domain (Oehring et al., 2012). The second combines an array of Kelch motifs with a GHKL (gyrase, Hsp90, histidine kinase, MutL)-type ATPase domain, which is a hallmark of microrchidia (MORC) proteins (Iyer et al., 2008). EELM2 and MORC proteins are typically found in a chromatin regulatory complex with histone deacetylase (HDAC) and nucleosome remodeling activities (Solari et al., 1999). Consistently, the *Toxoplasma gondii* ortholog of *Plasmodium* MORC forms part of a co-repressor complex with TgHDAC3 (Saksouk et al., 2005). In our interactome, MORC has a direct link with HDAC1, with a second EELM2 protein and with two other ApiAP2 proteins (Figure 3D; Table S5). These data lead us to hypothesize that *Plasmodium* MORC and EELM2 proteins form a scaffold connecting transcription factor complexes with epigenetic regulators to effect nucleosome reorganization and regulate gene expression. In support of such a mechanism, a DNA motif recognized by PF3D7_1107800, the *P. falciparum* ortholog of PBANKA_0939100, mirrors nucleosome spacing around transcriptional start sites (Kensche et al., 2016). Furthermore, the same ApiAP2 protein targets a genetic element in the introns of *P. falciparum var* genes that is important for tethering

members of this multigene family to the nuclear periphery as part of their epigenetic silencing (Zhang et al., 2011).

Affinity purification followed by MS analysis of HA-tagged SNF2L, a putative chromatin remodeling protein, identified uncharacterized protein PBANKA_1461600, confirming a physical link between these 2 proteins (Figures 3C and 3D), which are connected in the network through the eIF3 complex. We also confirmed a physical association between putative cohesin subunits SMC3 (PBANKA_0716000) and SMC1 (PBANKA_0917500) and identified a further SMC3-associated protein, uncharacterized protein PBANKA_1304000, which in the network is connected to both SMC1 and SMC3 through another uncharacterized protein that we did not detect in the pull-downs. PBANKA_1304000 contains an Rad21/Rec8-like N-terminal domain, which is a conserved N-terminal region present in eukaryotic cohesins. Our results indicate that PBANKA_1304000 is a subunit of the cohesin complex. These data demonstrate the utility of our interaction network in informing the biological function of uncharacterized *Plasmodium* proteins and validate the use of the BN-PAGE-based approach to identify protein interactions at a proteome scale.

Species-Specific Interactions and a Core Conserved *Plasmodium* Interactome

We took advantage of the three *Plasmodium* species datasets and carried out cross-species analysis, focusing on proteins specific to only one species or to a subset of *Plasmodium* species, which possibly affect important differential phenotypes between malaria parasites, such as their ability to cause human disease (Frech and Chen, 2011). Primate parasite proteins that are absent in rodent parasites could represent pathogenicity genes required for human infection, which potentially makes them interesting targets for the development of antimalarials (Frech and Chen, 2011; Müller and Kappes, 2007; Pessi et al., 2004). We found six examples of such proteins distributed in nine clusters (three of them overlapping). In agreement with their parasite specificity, none of their interactions were observed in *P. berghei* samples (Table S6). Three of these human *Plasmodium* specific proteins are discussed further below.

Cluster 7 contained phosphomethylpyrimidine kinase, a key enzyme of the vitamin B1 biosynthesis pathway. Rodent parasites lack three of the enzymes of the pathway, suggesting that they are incapable of *de novo* thiamin synthesis (Frech and Chen, 2011; Müller and Kappes, 2007). Thiamin metabolism has been proposed as antimalarial drug target (Chan et al., 2013). Cluster 7 also contained two uncharacterized *P. knowlesi* proteins without orthologs in other species, PKNH_1402300 and PKNH_1473300. The latter is a *Plasmodium* exported protein of the PHIST (*Plasmodium* helical interspersed subtelomeric family) family, whose members are present in many copies in human *Plasmodium* but only one in rodent *Plasmodium*, suggesting that cluster 7 is a primate-specific cluster potentially involved in the biosynthesis of thiamin.

Cluster 32 included primate-specific uncharacterized protein PF3D7_1118000 (Frech and Chen, 2011), *P. knowlesi* PIR (protein with interspersed repeats) family protein PKNH_1000600, and signal recognition particle (SRP) subunit SRP19, which is involved in SRP-dependent co-translational protein targeting to membranes. PIR protein PKNH_1000600 is part of the vir family. VIR proteins can localize to the membrane of infected erythrocytes and have been linked to immune evasion (Ben Mamoun et al., 2010; Bernabeu et al., 2012; del Portillo et al., 2001; Kilian et al., 2018). In light of its interactions, we hypothesize that PF3D7_1118000 may have a function related to endothelial adherence specifically in the human host.

Cluster 223 included phosphoethanolamine *N*-methyltransferase (PMT), an enzyme involved in polyamine and phospholipid metabolism that is absent in rodent *Plasmodium*, leading to marked differences in phospholipid biosynthetic pathways between human and rodent malaria parasites (Déchamps et al., 2010; Frech and Chen, 2011). Other proteins in the cluster were a putative hydrolase, a coatomer subunit, Fe-S cluster assembly factor NBP35, and PF3D7_1441100, a functionally uncharacterized protein annotated to the mitochondrial disulfide relay system metabolic pathway (PlasmoDB). Isoprenoid biosynthesis is predicted to rely on Fe-S cluster cofactors, but little is known about Fe-S cluster synthesis or the roles that Fe-S cluster proteins play in *Plasmodium* biology (Gisselberg et al., 2013). *P. falciparum* PMT is important for membrane biogenesis and parasite development, survival, and propagation; therefore, this and other enzymes in the phosphatidylcholine biosynthetic pathway are attractive targets for antimalarial therapy (Ben Mamoun et al., 2010; Kilian et al., 2018). In fact, all of the proteins in cluster 223 are deemed to be essential (Zhang et al., 2018).

To define a set of interactions conserved across the full range of the *Plasmodium* genus, we segregated edges that were common to protein families across the 3 species (7,117) and performed a cluster analysis. This identified 158 clusters conserved across all 3 *Plasmodium* species (Figures 4A and 4B; Table S9). These conserved clusters represent 50 of the 89 CORUM clusters detected in the high-confidence PPI network. This represents the most confident subset of interactions, since they were detected in biochemical fractionations in all three different *Plasmodium* species. We observed positive enrichment for "essential" and negative enrichment for "dispensable" proteins in this group (Fisher's exact test with Bonferroni correction, $p < 0.05$), using growth phenotype data from a recent large-scale *P. berghei* knockout screen (Bushell et al., 2017), indicating that these proteins are involved in core biological processes. To confirm our suggestion that these interactions are conserved, we compared the distribution of protein family relative evolutionary rates. We observed similar distributions for the protein families in each network, but slower relative evolutionary rates in the interactions conserved across species (Figure 4C). This confirms that the common protein interaction network embodies an evolutionarily conserved core set of protein interactions representing the essential backbone of *Plasmodium* schizont biology.

DISCUSSION

More than one-third of *Plasmodium* proteins lack functional annotation; elucidating the function of these proteins is crucial to understanding the biology of this divergent eukaryote of great medical importance. Predicting protein interactions reliably and at scale provides a means of assigning putative functions to uncharacterized proteins by associating them with other proteins of known function. While a plethora of protein interaction data exists for model organisms such as yeast, fly, and mouse,

OPEN
ACCESS
CellPress

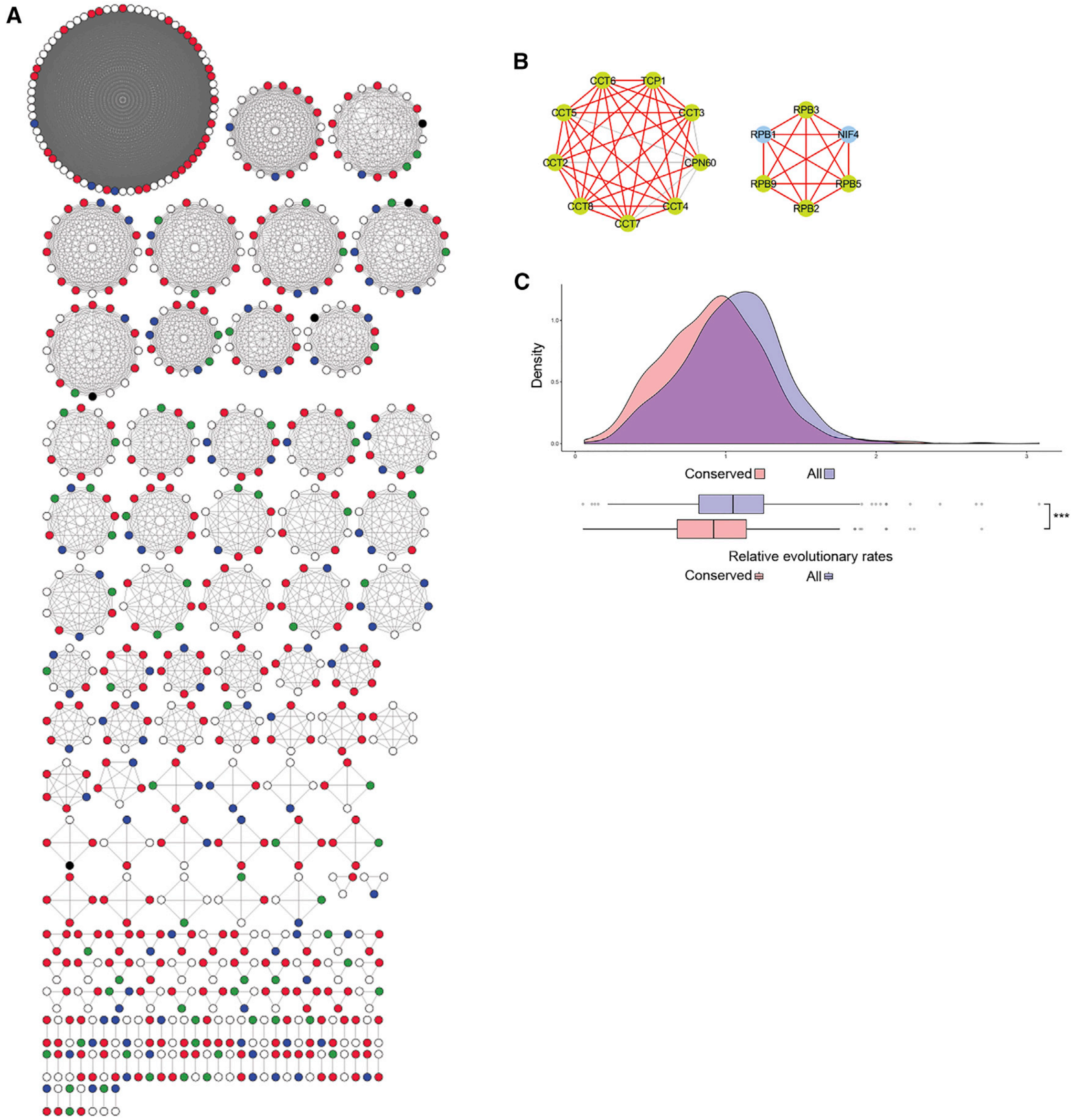

Figure 4. A Conserved Malaria Interaction Network

(A) Clusters were generated from the protein interactions common to all three *Plasmodium* species studied, visualized as network graphs. The essential proteins are shown as red nodes, the blue nodes are proteins whose absence leads to slow growth, and the green nodes are proteins that cause no growth phenotype when absent.

(B) Examples of conserved clusters detected in all three *Plasmodium* species. Interactions between the green nodes were detected in all of the species; the interactions with the blue nodes were not detected in *P. knowlesi*. The red edges represent the interactions annotated in STRING.

(C) Density distribution and boxplots of relative evolutionary rates of OrthoMCL protein families to *P. falciparum*. The plots for the high-confidence PPI network and of protein families in the clusters from (A) are shown. The differences in distribution were assessed with a Wilcoxon rank-sum test (***$p < 2.9e{-14}$).

See also Table S9.

and human cells, such information is sparse in *Plasmodium*. Most interactions reported in databases such as STRING for *Plasmodium* proteins in fact derive from experimental evidence obtained for orthologous proteins in other organisms. Previous experimental evidence of protein interactions in *Plasmodium* primarily come from *ad hoc* studies of a very small number of proteins. The only large-scale interaction study to date, a yeast two-hybrid screen (LaCount et al., 2005), has had limited impact, and the difficulty of expressing sufficiently large domains of very AT-rich parasite genes in yeast suggests that it may contain high numbers of both false-positives and false-negatives.

The biochemically determined interaction data presented here encompassing 20,000 associations between almost 2,000 *Plasmodium* proteins provide functional information for >200 non-conserved parasite proteins with no previous annotations. Other large-scale interaction studies based on biochemical

fractionation have used size exclusion, ion exchange chromatography, or density gradient. The BN-PAGE separation used here provides an alternative strategy with a successful record in dealing with membrane proteins that can be implemented without the need for specialist chromatographic equipment. Our data provide a significant advance in the understanding of *Plasmodium* biology and represent an invaluable resource for the malaria research community to build on and integrate additional experimental and computational data.

The fact that we are able to make detailed predictions that can be validated experimentally through affinity purification coupled to MS, the current method of choice for determining protein interactions (although the two interaction mapping methods, BN comigration and affinity purification, are based on different biochemical attributes) highlights the usefulness of the work presented here. We confirm several interactions involving three candidate targets, in addition to identifying unreported associations. It should be noted that the selection of candidates was made from a predetermined list of available tagged proteins, not necessarily from the most confident interactions in the network. The validation data confirming the link of ApiAP2 transcription factors to epigenetic regulation via MORC and EELM2 give rise to tantalizing hypotheses regarding the mechanisms through which these key regulators of dynamic gene expression programs during parasite development act. This demonstrates the power of our study to inform and guide future mechanistic research of specific parasite cellular processes.

We have made the interaction dataset available in *Plasmo*GEM (https://plasmogem.shinyapps.io/schizont_interactions/) as a searchable database allowing easy access. To facilitate the use and navigation of the interaction network and to allow the user to evaluate the strength of the evidence for each interaction, we also provided all of the underlying data (including RF analysis score, correlation scores, supporting features, and attributes of the proteins) in consolidated files that are compatible with network visualization programs, allowing interactive display and exploration of the malaria interactome (Tables S4, S5, and S6). These tables enable a review of the scores and the supporting features for protein pairs of interest used for determining an interaction and the attributes of proteins involved, and they can assist with the prioritization of candidate interactions for further studies.

Summing up, the data presented here demonstrate that systems-level BN-PAGE fractionation studies coupled to protein correlation profiling (GBC-MS) provide a valuable complement to previous interactome studies based on chromatographic fractionation, which have mainly focused on soluble complexes. Our integrative interaction proteomics approach has resulted in the identification of >20,000 interactions encompassing one-third of the malaria proteome, yielding the broadest experimentally determined malaria interactome available. This represents a rich hypothesis-generating resource that will be the basis of much future work. Information derived from our PPI network will be useful to assign function to >200 uncharacterized *Plasmodium* proteins; provide mechanistic insight into the roles of proteins, guide directed studies on the function, structure, and dynamics of malaria proteins; and help characterize potential therapeutic targets. This *Plasmodium* schizont interactome paves the way for investigating changes in protein interactions during the different phases of the malaria parasite life cycle at the systems level.

STAR★METHODS

Detailed methods are provided in the online version of this paper and include the following:

- KEY RESOURCES TABLE
- LEAD CONTACT AND MATERIALS AVAILABILITY
- EXPERIMENTAL MODEL AND SUBJECT DETAILS
- METHOD DETAILS
  - Isolation of *Plasmodium* schizonts
  - Blue native PAGE of schizont lysates
  - In-gel protein digestion
  - P. berghei transfections
  - HA affinity purification for MS analysis
  - Liquid chromatography-Tandem mass spectrometry
  - Analysis of mass spectrometry data
- QUANTIFICATION AND STATISTICAL ANALYSIS
  - Bioinformatics Analysis
- DATA AND CODE AVAILABILITY
- ADDITIONAL RESOURCES

SUPPLEMENTAL INFORMATION

Supplemental Information can be found online at https://doi.org/10.1016/j.celrep.2019.07.019.

ACKNOWLEDGMENT

This work was supported by the Wellcome Trust (grant no. 206194/Z/17/Z).

AUTHOR CONTRIBUTIONS

Conceptualization, J.S.C., J.C.R., M.P., and O.B. Formal Analysis, C. Hillier, M.P., and O.B. Investigation, C. Hillier, C. Herd, E.B., M.P., T.M., and T.S. Resources, B.A. Writing – Original Draft, C. Hillier and M.P. Writing – Review & Editing, M.P., C. Hillier, E.B., J.S.C., J.C.R., O.B., and T.S. Visualization, C. Hillier, M.P., and O.B. Supervision, E.B., J.S.C., J.C.R., M.P., and O.B.

DECLARATION OF INTERESTS

The authors declare no competing interests.

Received: December 13, 2018
Revised: May 28, 2019
Accepted: July 8, 2019
Published: August 6, 2019

REFERENCES

Aurrecoechea, C., Brestelli, J., Brunk, B.P., Dommer, J., Fischer, S., Gajria, B., Gao, X., Gingle, A., Grant, G., Harb, O.S., et al. (2009). PlasmoDB: a functional genomic database for malaria parasites. Nucleic Acids Res. *37*, D539–D543.

Balaji, S., Babu, M.M., Iyer, L.M., and Aravind, L. (2005). Discovery of the principal specific transcription factors of Apicomplexa and their implication for the evolution of the AP2-integrase DNA binding domains. Nucleic Acids Res. *33*, 3994–4006.

Ben Mamoun, C., Prigge, S.T., and Vial, H. (2010). Targeting the Lipid Metabolic Pathways for the Treatment of Malaria. Drug Dev. Res. *71*, 44–55.

Bernabeu, M., Lopez, F.J., Ferrer, M., Martin-Jaular, L., Razaname, A., Corradin, G., Maier, A.G., Del Portillo, H.A., and Fernandez-Becerra, C. (2012). Functional analysis of Plasmodium vivax VIR proteins reveals different subcellular localizations and cytoadherence to the ICAM-1 endothelial receptor. Cell. Microbiol. *14*, 386–400.

OPEN ACCESS
CellPress

Bode, D., Yu, L., Tate, P., Pardo, M., and Choudhary, J. (2016). Characterization of Two Distinct Nucleosome Remodeling and Deacetylase (NuRD) Complex Assemblies in Embryonic Stem Cells. Mol. Cell. Proteomics *15*, 878–891.

Bozdech, Z., Llinás, M., Pulliam, B.L., Wong, E.D., Zhu, J., and DeRisi, J.L. (2003). The transcriptome of the intraerythrocytic developmental cycle of Plasmodium falciparum. PLoS Biol. *1*, E5.

Bushell, E., Gomes, A.R., Sanderson, T., Anar, B., Girling, G., Herd, C., Metcalf, T., Modrzynska, K., Schwach, F., Martin, R.E., et al. (2017). Functional Profiling of a Plasmodium Genome Reveals an Abundance of Essential Genes. Cell *170*, 260–272.e8.

Chan, X.W., Wrenger, C., Stahl, K., Bergmann, B., Winterberg, M., Müller, I.B., and Saliba, K.J. (2013). Chemical and genetic validation of thiamine utilization as an antimalarial drug target. Nat. Commun. *4*, 2060.

Cowman, A.F., Healer, J., Marapana, D., and Marsh, K. (2016). Malaria: Biology and Disease. Cell *167*, 610–624.

Cox, J., and Mann, M. (2008). MaxQuant enables high peptide identification rates, individualized p.p.b.-range mass accuracies and proteome-wide protein quantification. Nat. Biotechnol. *26*, 1367–1372.

Crozier, T.W.M., Tinti, M., Larance, M., Lamond, A.I., and Ferguson, M.A.J. (2017). Prediction of Protein Complexes in *Trypanosoma brucei* by Protein Correlation Profiling Mass Spectrometry and Machine Learning. Mol. Cell. Proteomics *16*, 2254–2267.

de Koning-Ward, T.F., Gilson, P.R., Boddey, J.A., Rug, M., Smith, B.J., Papenfuss, A.T., Sanders, P.R., Lundie, R.J., Maier, A.G., Cowman, A.F., and Crabb, B.S. (2009). A newly discovered protein export machine in malaria parasites. Nature *459*, 945–949.

Déchamps, S., Maynadier, M., Wein, S., Gannoun-Zaki, L., Maréchal, E., and Vial, H.J. (2010). Rodent and nonrodent malaria parasites differ in their phospholipid metabolic pathways. J. Lipid Res. *51*, 81–96.

del Portillo, H.A., Fernandez-Becerra, C., Bowman, S., Oliver, K., Preuss, M., Sanchez, C.P., Schneider, N.K., Villalobos, J.M., Rajandream, M.A., Harris, D., et al. (2001). A superfamily of variant genes encoded in the subtelomeric region of Plasmodium vivax. Nature *410*, 839–842.

Dharmarajan, V., Lee, J.H., Patel, A., Skalnik, D.G., and Cosgrove, M.S. (2012). Structural basis for WDR5 interaction (Win) motif recognition in human SET1 family histone methyltransferases. J. Biol. Chem. *287*, 27275–27289.

Dou, Y., Milne, T.A., Tackett, A.J., Smith, E.R., Fukuda, A., Wysocka, J., Allis, C.D., Chait, B.T., Hess, J.L., and Roeder, R.G. (2005). Physical association and coordinate function of the H3 K4 methyltransferase MLL1 and the H4 K16 acetyltransferase MOF. Cell *121*, 873–885.

Edgar, R.C. (2004a). MUSCLE: a multiple sequence alignment method with reduced time and space complexity. BMC Bioinformatics *5*, 113.

Edgar, R.C. (2004b). MUSCLE: multiple sequence alignment with high accuracy and high throughput. Nucleic Acids Res. *32*, 1792–1797.

Frech, C., and Chen, N. (2011). Genome comparison of human and non-human malaria parasites reveals species subset-specific genes potentially linked to human disease. PLoS Comput. Biol. *7*, e1002320.

Frénal, K., Dubremetz, J.F., Lebrun, M., and Soldati-Favre, D. (2017). Gliding motility powers invasion and egress in Apicomplexa. Nat. Rev. Microbiol. *15*, 645–660.

Gavin, A.C., Bösche, M., Krause, R., Grandi, P., Marzioch, M., Bauer, A., Schultz, J., Rick, J.M., Michon, A.M., Cruciat, C.M., et al. (2002). Functional organization of the yeast proteome by systematic analysis of protein complexes. Nature *415*, 141–147.

Gavin, A.C., Aloy, P., Grandi, P., Krause, R., Boesche, M., Marzioch, M., Rau, C., Jensen, L.J., Bastuck, S., Dümpelfeld, B., et al. (2006). Proteome survey reveals modularity of the yeast cell machinery. Nature *440*, 631–636.

Gisselberg, J.E., Dellibovi-Ragheb, T.A., Matthews, K.A., Bosch, G., and Prigge, S.T. (2013). The suf iron-sulfur cluster synthesis pathway is required for apicoplast maintenance in malaria parasites. PLoS Pathog. *9*, e1003655.

Hall, N., Karras, M., Raine, J.D., Carlton, J.M., Kooij, T.W., Berriman, M., Florens, L., Janssen, C.S., Pain, A., Christophides, G.K., et al. (2005). A comprehensive survey of the Plasmodium life cycle by genomic, transcriptomic, and proteomic analyses. Science *307*, 82–86.

Havugimana, P.C., Hart, G.T., Nepusz, T., Yang, H., Turinsky, A.L., Li, Z., Wang, P.I., Boutz, D.R., Fong, V., Phanse, S., et al. (2012). A census of human soluble protein complexes. Cell *150*, 1068–1081.

Heide, H., Bleier, L., Steger, M., Ackermann, J., Dröse, S., Schwamb, B., Zörnig, M., Reichert, A.S., Koch, I., Wittig, I., and Brandt, U. (2012). Complexome profiling identifies TMEM126B as a component of the mitochondrial complex I assembly complex. Cell Metab. *16*, 538–549.

Hein, M.Y., Hubner, N.C., Poser, I., Cox, J., Nagaraj, N., Toyoda, Y., Gak, I.A., Weisswange, I., Mansfeld, J., Buchholz, F., et al. (2015). A human interactome in three quantitative dimensions organized by stoichiometries and abundances. Cell *163*, 712–723.

Hu, G., Cabrera, A., Kono, M., Mok, S., Chaal, B.K., Haase, S., Engelberg, K., Cheemadan, S., Spielmann, T., Preiser, P.R., et al. (2010). Transcriptional profiling of growth perturbations of the human malaria parasite Plasmodium falciparum. Nat. Biotechnol. *28*, 91–98.

Huttlin, E.L., Ting, L., Bruckner, R.J., Gebreab, F., Gygi, M.P., Szpyt, J., Tam, S., Zarraga, G., Colby, G., Baltier, K., et al. (2015). The BioPlex Network: A Systematic Exploration of the Human Interactome. Cell *162*, 425–440.

Huttlin, E.L., Bruckner, R.J., Paulo, J.A., Cannon, J.R., Ting, L., Baltier, K., Colby, G., Gebreab, F., Gygi, M.P., Parzen, H., et al. (2017). Architecture of the human interactome defines protein communities and disease networks. Nature *545*, 505–509.

Iyer, L.M., Anantharaman, V., Wolf, M.Y., and Aravind, L. (2008). Comparative genomics of transcription factors and chromatin proteins in parasitic protists and other eukaryotes. Int. J. Parasitol. *38*, 1–31.

Janse, C.J., Franke-Fayard, B., and Waters, A.P. (2006). Selection by flow-sorting of genetically transformed, GFP-expressing blood stages of the rodent malaria parasite, Plasmodium berghei. Nat. Protoc. *1*, 614–623.

Juan, D., Pazos, F., and Valencia, A. (2008). High-confidence prediction of global interactomes based on genome-wide coevolutionary networks. Proc. Natl. Acad. Sci. USA *105*, 934–939.

Kaderi Kibria, K.M., Rawat, K., Klinger, C.M., Datta, G., Panchal, M., Singh, S., Iyer, G.R., Kaur, I., Sharma, V., Dacks, J.B., et al. (2015). A role for adaptor protein complex 1 in protein targeting to rhoptry organelles in Plasmodium falciparum. Biochim. Biophys. Acta *1853*, 699–710.

Kastritis, P.L., O'Reilly, F.J., Bock, T., Li, Y., Rogon, M.Z., Buczak, K., Romanov, N., Betts, M.J., Bui, K.H., Hagen, W.J., et al. (2017). Capturing protein communities by structural proteomics in a thermophilic eukaryote. Mol. Syst. Biol. *13*, 936.

Kensche, P.R., Hoeijmakers, W.A., Toenhake, C.G., Bras, M., Chappell, L., Berriman, M., and Bártfai, R. (2016). The nucleosome landscape of Plasmodium falciparum reveals chromatin architecture and dynamics of regulatory sequences. Nucleic Acids Res. *44*, 2110–2124.

Kilian, N., Choi, J.Y., Voelker, D.R., and Ben Mamoun, C. (2018). Role of phospholipid synthesis in the development and differentiation of malaria parasites in the blood. J. Biol. Chem. *293*, 17308–17316.

Kirkwood, K.J., Ahmad, Y., Larance, M., and Lamond, A.I. (2013). Characterization of native protein complexes and protein isoform variation using size-fractionation-based quantitative proteomics. Mol. Cell. Proteomics *12*, 3851–3873.

Kristensen, A.R., Gsponer, J., and Foster, L.J. (2012). A high-throughput approach for measuring temporal changes in the interactome. Nat. Methods *9*, 907–909.

LaCount, D.J., Vignali, M., Chettier, R., Phansalkar, A., Bell, R., Hesselberth, J.R., Schoenfeld, L.W., Ota, I., Sahasrabudhe, S., Kurschner, C., et al. (2005). A protein interaction network of the malaria parasite Plasmodium falciparum. Nature *438*, 103–107.

Liaw, A., and Wiener, M. (2001). Classification and Regression by RandomForest. R News *2/3*, 18–22.

Logan-Klumpler, F.J., De Silva, N., Boehme, U., Rogers, M.B., Velarde, G., McQuillan, J.A., Carver, T., Aslett, M., Olsen, C., Subramanian, S., et al.

(2012). GeneDB–an annotation database for pathogens. Nucleic Acids Res. *40*, D98–D108.

Modrzynska, K., Pfander, C., Chappell, L., Yu, L., Suarez, C., Dundas, K., Gomes, A.R., Goulding, D., Rayner, J.C., Choudhary, J., and Billker, O. (2017). A Knockout Screen of ApiAP2 Genes Reveals Networks of Interacting Transcriptional Regulators Controlling the Plasmodium Life Cycle. Cell Host Microbe *21*, 11–22.

Moon, R.W., Hall, J., Rangkuti, F., Ho, Y.S., Almond, N., Mitchell, G.H., Pain, A., Holder, A.A., and Blackman, M.J. (2013). Adaptation of the genetically tractable malaria pathogen Plasmodium knowlesi to continuous culture in human erythrocytes. Proc. Natl. Acad. Sci. USA *110*, 531–536.

Müller, S., and Kappes, B. (2007). Vitamin and cofactor biosynthesis pathways in Plasmodium and other apicomplexan parasites. Trends Parasitol. *23*, 112–121.

Nepusz, T., Yu, H., and Paccanaro, A. (2012). Detecting overlapping protein complexes in protein-protein interaction networks. Nat. Methods *9*, 471–472.

Ochoa, D., Juan, D., Valencia, A., and Pazos, F. (2015). Detection of significant protein coevolution. Bioinformatics *31*, 2166–2173.

Oehring, S.C., Woodcroft, B.J., Moes, S., Wetzel, J., Dietz, O., Pulfer, A., Dekiwadia, C., Maeser, P., Flueck, C., Witmer, K., et al. (2012). Organellar proteomics reveals hundreds of novel nuclear proteins in the malaria parasite Plasmodium falciparum. Genome Biol. *13*, R108.

Owen, D.J., Ornaghi, P., Yang, J.C., Lowe, N., Evans, P.R., Ballario, P., Neuhaus, D., Filetici, P., and Travers, A.A. (2000). The structural basis for the recognition of acetylated histone H4 by the bromodomain of histone acetyltransferase gcn5p. EMBO J. *19*, 6141–6149.

Pardo, M., Lang, B., Yu, L., Prosser, H., Bradley, A., Babu, M.M., and Choudhary, J. (2010). An expanded Oct4 interaction network: implications for stem cell biology, development, and disease. Cell Stem Cell *6*, 382–395.

Pardo, M., Bode, D., Yu, L., and Choudhary, J.S. (2017). Resolving Affinity Purified Protein Complexes by Blue Native PAGE and Protein Correlation Profiling. J. Vis. Exp. *122*.

Pessi, G., Kociubinski, G., and Mamoun, C.B. (2004). A pathway for phosphatidylcholine biosynthesis in Plasmodium falciparum involving phosphoethanolamine methylation. Proc. Natl. Acad. Sci. USA *101*, 6206–6211.

Pfeffer, S.R., Dirac-Svejstrup, A.B., and Soldati, T. (1995). Rab GDP dissociation inhibitor: putting rab GTPases in the right place. J. Biol. Chem. *270*, 17057–17059.

Pradel, G., Hayton, K., Aravind, L., Iyer, L.M., Abrahamsen, M.S., Bonawitz, A., Mejia, C., and Templeton, T.J. (2004). A multidomain adhesion protein family expressed in Plasmodium falciparum is essential for transmission to the mosquito. J. Exp. Med. *199*, 1533–1544.

Raghavachari, B., Tasneem, A., Przytycka, T.M., and Jothi, R. (2008). DOMINE: a database of protein domain interactions. Nucleic Acids Res. *36*, D656–D661.

Ramaprasad, A., Pain, A., and Ravasi, T. (2012). Defining the protein interaction network of human malaria parasite Plasmodium falciparum. Genomics *99*, 69–75.

Reid, A.J., Talman, A.M., Bennett, H.M., Gomes, A.R., Sanders, M.J., Illingworth, C.J.R., Billker, O., Berriman, M., and Lawniczak, M.K. (2018). Single-cell RNA-seq reveals hidden transcriptional variation in malaria parasites. eLife *7*, e33105.

Revenko, A.S., Kalashnikova, E.V., Gemo, A.T., Zou, J.X., and Chen, H.W. (2010). Chromatin loading of E2F-MLL complex by cancer-associated coregulator ANCCA via reading a specific histone mark. Mol. Cell. Biol. *30*, 5260–5272.

Saeed, S., Carter, V., Tremp, A.Z., and Dessens, J.T. (2010). Plasmodium berghei crystalloids contain multiple LCCL proteins. Mol. Biochem. Parasitol. *170*, 49–53.

Saksouk, N., Bhatti, M.M., Kieffer, S., Smith, A.T., Musset, K., Garin, J., Sullivan, W.J., Jr., Cesbron-Delauw, M.F., and Hakimi, M.A. (2005). Histone-modifying complexes regulate gene expression pertinent to the differentiation of the protozoan parasite Toxoplasma gondii. Mol. Cell. Biol. *25*, 10301–10314.

Schägger, H., and von Jagow, G. (1991). Blue native electrophoresis for isolation of membrane protein complexes in enzymatically active form. Anal. Biochem. *199*, 223–231.

Schägger, H., Cramer, W.A., and von Jagow, G. (1994). Analysis of molecular masses and oligomeric states of protein complexes by blue native electrophoresis and isolation of membrane protein complexes by two-dimensional native electrophoresis. Anal. Biochem. *217*, 220–230.

Shannon, P., Markiel, A., Ozier, O., Baliga, N.S., Wang, J.T., Ramage, D., Amin, N., Schwikowski, B., and Ideker, T. (2003). Cytoscape: a software environment for integrated models of biomolecular interaction networks. Genome Res. *13*, 2498–2504.

Simon, N., Scholz, S.M., Moreira, C.K., Templeton, T.J., Kuehn, A., Dude, M.A., and Pradel, G. (2009). Sexual stage adhesion proteins form multi-protein complexes in the malaria parasite Plasmodium falciparum. J. Biol. Chem. *284*, 14537–14546.

Solari, F., Bateman, A., and Ahringer, J. (1999). The Caenorhabditis elegans genes egl-27 and egr-1 are similar to MTA1, a member of a chromatin regulatory complex, and are redundantly required for embryonic patterning. Development *126*, 2483–2494.

Szklarczyk, D., Morris, J.H., Cook, H., Kuhn, M., Wyder, S., Simonovic, M., Santos, A., Doncheva, N.T., Roth, A., Bork, P., et al. (2017). The STRING database in 2017: quality-controlled protein-protein association networks, made broadly accessible. Nucleic Acids Res. *45* (*D1*), D362–D368.

Teo, G., Liu, G., Zhang, J., Nesvizhskii, A.I., Gingras, A.C., and Choi, H. (2014). SAINTexpress: improvements and additional features in Significance Analysis of INTeractome software. J. Proteomics *100*, 37–43.

Trager, W., and Jensen, J.B. (1976). Human malaria parasites in continuous culture. Science *193*, 673–675.

Treeck, M., Sanders, J.L., Elias, J.E., and Boothroyd, J.C. (2011). The phosphoproteomes of Plasmodium falciparum and Toxoplasma gondii reveal unusual adaptations within and beyond the parasites' boundaries. Cell Host Microbe *10*, 410–419.

Tyanova, S., Temu, T., Sinitcyn, P., Carlson, A., Hein, M.Y., Geiger, T., Mann, M., and Cox, J. (2016). The Perseus computational platform for comprehensive analysis of (prote)omics data. Nat. Methods *13*, 731–740.

Veerassamy, S., Smith, A., and Tillier, E.R. (2003). A transition probability model for amino acid substitutions from blocks. J. Comput. Biol. *10*, 997–1010.

Vizcaíno, J.A., Csordas, A., Del-Toro, N., Dianes, J.A., Griss, J., Lavidas, I., Mayer, G., Perez-Riverol, Y., Reisinger, F., Ternent, T., et al. (2016). 2016 update of the PRIDE database and its related tools. Nucleic Acids Res. *44*, 11033.

Wan, C., Borgeson, B., Phanse, S., Tu, F., Drew, K., Clark, G., Xiong, X., Kagan, O., Kwan, J., Bezginov, A., et al. (2015). Panorama of ancient metazoan macromolecular complexes. Nature *525*, 339–344.

World Health Organization (2017). World malaria report 2017. https://apps.who.int/iris/bitstream/handle/10665/259492/9789241565523-eng.pdf.

Yellaboina, S., Tasneem, A., Zaykin, D.V., Raghavachari, B., and Jothi, R. (2011). DOMINE: a comprehensive collection of known and predicted domain-domain interactions. Nucleic Acids Res. *39*, D730–D735.

Zeeshan, M., Kaur, I., Joy, J., Saini, E., Paul, G., Kaushik, A., Dabral, S., Mohmmed, A., Gupta, D., and Malhotra, P. (2017). Proteomic Identification and Analysis of Arginine-Methylated Proteins of Plasmodium falciparum at Asexual Blood Stages. J. Proteome Res. *16*, 368–383.

Zhang, Q., Huang, Y., Zhang, Y., Fang, X., Claes, A., Duchateau, M., Namane, A., Lopez-Rubio, J.J., Pan, W., and Scherf, A. (2011). A critical role of perinuclear filamentous actin in spatial repositioning and mutually exclusive expression of virulence genes in malaria parasites. Cell Host Microbe *10*, 451–463.

Zhang, M., Wang, C., Otto, T.D., Oberstaller, J., Liao, X., Adapa, S.R., Udenze, K., Bronner, I.F., Casandra, D., Mayho, M., et al. (2018). Uncovering the essential genes of the human malaria parasite *Plasmodium falciparum* by saturation mutagenesis. Science *360*, eaap7847.

Zhu, P., Zhou, W., Wang, J., Puc, J., Ohgi, K.A., Erdjument-Bromage, H., Tempst, P., Glass, C.K., and Rosenfeld, M.G. (2007). A histone H2A deubiquitinase complex coordinating histone acetylation and H1 dissociation in transcriptional regulation. Mol. Cell *27*, 609–621.

STAR★METHODS

KEY RESOURCES TABLE

REAGENT or RESOURCE	SOURCE	IDENTIFIER
Antibodies		
Anti-HA High Affinity, Rat monoclonal antibody (clone 3F10)	Roche	Cat#11867423001; RRID:AB_390918
Critical Commercial Assays		
NativePAGE Sample Buffer (4X)	Life Technologies	Cat#BN2003
NativePAGE 5% G-250 Sample Additive	Life Technologies	Cat#BN2004
NativePAGE Running Buffer (20X)	Life Technologies	Cat#BN2001
NativePAGE 3-12% Bis-Tris gels	Life Technologies	Cat#BN1001
NativePAGE Cathode Buffer Additive (20X)	Life Technologies	Cat#BN2002
Deposited Data		
Raw mass spectrometry data	This paper	https://www.ebi.ac.uk/pride/archive/ (identifiers PXD009039, PXD010117)
Experimental Models: Organisms/Strains		
Plasmodium falciparum strain 3D7	N/A	3D7
RCC Han Wistar outbred rat(female, 8 week)	Envigo+++	RccHan:WIST
Theiler's original (TO) outbred mouse (female, 8-12 week)	Envigo+++	HsdOla:TO
Plasmodium berghei ANKA cl15cy1	N/A	cl15cy1
Plasmodium knowlesi clone A1-H.1	N/A	A1-H.1
Recombinant DNA		
P. berghei 3xHA tagging vector for PBANKA_093910	https://plasmogem.sanger.ac.uk	PbGEM-094925
P. berghei 3xHA tagging vector for PBANKA_071600	https://plasmogem.sanger.ac.uk	PbGEM-281970
P. berghei 3xHA tagging vector for PBANKA_094270	https://plasmogem.sanger.ac.uk	PbGEM-292320
Software and Algorithms		
Cytoscape	https://cytoscape.org/	3.4.0
MaxQuant	https://www.biochem.mpg.de/5111795/maxquant	version 1.5.5.1
Mascot	Matrix Science	2.4
R	https://www.r-project.org	N/A
Perseus	https://www.biochem.mpg.de/5111810/perseus	1.6.1.1

LEAD CONTACT AND MATERIALS AVAILABILITY

Further information and requests for resources and reagents should be directed to and will be fulfilled by the Lead Contact, Mercedes Pardo (Mercedes.pardocalvo@icr.ac.uk). All materials transferred will require Material Transfer Agreements (MTAs) to be arranged between the two institutions.

EXPERIMENTAL MODEL AND SUBJECT DETAILS

The *P. berghei* parasite line used was the cl15cy1 ANKA reference clone (Hall et al., 2005). All animal work was performed under licenses from the UK Home Office, with protocols approved by the Animal Welfare and Ethical Review Body of the Wellcome Sanger Institute. Rodents were reared in specific-pathogen-free conditions, and were monitored, housed and maintained as previously described (Bushell et al., 2017). Parasitaemia of infected animals were determined by light microscopy of Giemsa stained of thin blood smears.

Eight-twelve week old female Theiler's original (TO) outbred mice (Envigo, UK) were used as a donors for the *P. berghei* schizont cultures. This mouse strain was chosen to attain robust *P. berghei* infections with a low frequency of cerebral malaria. For transfections, an eight week old female RCC Han Wistar outbred rat (Envigo, UK) was utilized to generate parasites for the schizont culture.

Rats are used because they give rise to more schizonts with a higher transfection efficiency compared to mice. The very high transfection efficiency also means that no dilution cloning was required prior to commencing work with the 3xHA epitope tagged lines. Animals were infected via the intraperitoneal injection route, and on day two (mice) or five (rats) of infection at a parasitemia of ~1%–5%, the animals were terminally anaesthetised followed by cardiac puncture to collect the *P. berghei* infected blood. Following transfection parasites were injected intravenously into the tail vein of 8-12 week old female TO mice.

P. knowlesi clone A1-H.1 was maintained as previously described (Moon et al., 2013). *P. falciparum* strain 3D7 was cultured in RPMI-based media supplemented with 0.5% AlbuMAX II (Life Technologies), 2 mM L-glutamine and O+ human erythrocytes, using standard techniques as described (Trager and Jensen, 1976).

METHOD DETAILS

Isolation of *Plasmodium* schizonts

Mouse-derived *P. berghei* infected erythrocytes were put into culture for 22-24 hours to generate schizonts and these were purified at room temperature as described previously (Janse et al., 2006) with some modifications. Parasite blood cultures were checked for mature schizonts by giemsa staining. Leucocytes were removed from the culture by passing through a Plasmodipur filter (EuroProxima). Blood cultures were pelleted at 300 g for 14 min. Supernatants were removed leaving 3 mL behind and the pellets resuspended. The erythrocyte suspension was gently layered onto a Histodenz (Sigma) gradient and centrifuged at 300 g for 20 min. Schizont-infected erythrocytes were collected from the brown layer at the interface of the two suspensions and diluted in the supernatant from the parasite blood culture. Isolated schizont-infected erythrocytes were pelleted by centrifugation at 450 g for 3 min and washed twice with PBS.

P. falciparum schizonts were freed from erythrocytes by incubation with 0.1% saponin in PBS for 10 minutes and then washed with PBS to remove saponin.

P. knowlesi schizonts were purified by centrifugation at 1500 g for 10 minutes onto a 55% (w/v) Nycodenz cushion. They were then washed in RPMI-1640, and then freed from erythrocytes by incubation with 0.1% saponin in PBS, followed by a PBS wash.

Blue native PAGE of schizont lysates

Purified *Plasmodium* schizonts were lysed in 50 mM Tris pH 8, 150 mM NaCl, 1 mM EDTA, containing Halt protease and phosphatase inhibitor cocktail (Thermo Scientific), supplemented with 0, 0.1 or 1% NP-40, as previously described (Pardo et al., 2010). The cleared lysate was dialysed into 20 mM Bis-Tris pH 7, 500 mM 6-aminocaproic acid, 12 mM NaCl, 2 mM EDTA, containing Halt protease and phosphatase inhibitor cocktail, supplemented with 0 (for lysates with no NP-40) or 0.1% (for lysates with 0.1 or 1% NP-40), by centrifugal filtration through a 10 kDa cut-off membrane (PES, Vivaspin 500, Sartorius). Protein concentration was determined using the Bradford protein quantification assay.

The dialysed lysate containing 50 μg of protein was separated by Blue Native PAGE in NativePAGE 3%–12% Bis-Tris gels as previously described (Bode et al., 2016). Samples were prepared by addition of 4x native PAGE sample loading buffer (Life Technologies) and G-250 sample according to manufacturer's instructions. Gels were fixed in 40% methanol and 2% acetic acid for 30 minutes and then left in water until further processing.

In-gel protein digestion

Gel lanes were each excised into 48 1.5 mm-slices with a grid cutter (THISTLE Scientific) and these placed into a 96-well plate for further processing (Pardo et al., 2017). Proteins were reduced with 5 mM TCEP, followed by alkylation with 10 mM iodoacetamide. After complete gel destaining, proteins were digested with 1 ng/μL trypsin (sequencing grade, Roche). Peptide extraction was performed as described previously (Pardo et al., 2017). Peptide solutions were supplemented with acetonitrile to 60% final concentration, filtered through a 0.65 μm pore membrane plate (Multiscreen HTS DV, Millipore) to remove particulate material and dried. Peptides were resuspended in 0.4% formic acid and 80 mM ammonium bicarbonate and frozen until further analysis.

P. berghei transfections

All *P. berghei* 3xHA tagging vectors were obtained from the PlasmoGEM project and details of the constructs are available at https://plasmogem.sanger.ac.uk. The constructs transfected in this study were PbGEM-094925 (PBANKA_093910), PbGEM-281970 (PBANKA_071600) and PbGEM-292320 (PBANKA_094270). For each construct 1-2 μg of NotI-HF (New England Biolabs) digested DNA (MIDI prep, QIAGEN) was purified by standard ethanol precipitation prior to transfection. *P. berghei* schizonts were prepared by culturing 22 hours *ex vivo*, purified on a Histodenz gradient and transfected using the FI115 program on the Lonza 4D-Nucleofector core system (with the X Unit) together with the P3 Primary Cell 4D-Nucleofector solution (Lonza), as previously described (Bushell et al., 2017; Janse et al., 2006). Transfectant parasites were selected for and maintained under 0.07mg/mL pyrimethamine (Sigma) administered in drinking water, and were prepared for HA immunoprecipitation without prior dilution cloning.

HA affinity purification for MS analysis

Purified *Plasmodium* schizonts (wild-type or expressing an HA-tagged protein) were lysed in 50 mM Tris pH 8, 150 mM NaCl, 1 mM EDTA, containing Halt protease and phosphatase inhibitor cocktail (Thermo Scientific), supplemented with 0.1 or 1% NP-40, as

previously described (Pardo et al., 2010). Anti-HA antibody (Roche, 14 μg) was coupled to 100 μl of Protein G Dynabeads (Life Technologies). Cleared lysates containing 1 mg of total protein were incubated with anti-HA coupled Dynabeads for 2 hours at 4°C. After removing the supernatant beads were washed four times with IPP150 buffer (Pardo et al., 2010) and then 3 times with 50 mM ammonium bicarbonate. Beads were then resuspended in 50 mM ammonium bicarbonate and 1 μg of trypsin (sequencing grade, Roche) was added. Digestion was carried out at 37°C overnight with constant shaking. Peptide solutions were recovered from the beads, supplemented with acetonitrile to 60% final concentration and filtered through a 0.65 μm pore membrane plate (Multiscreen HTS DV, Millipore). Peptides were then dried, reduced with 40 mM TCEP for 15 minutes at room temperature, acidified with formic acid at 0.5% final concentration and frozen until further analysis. HA (baits and control) AP-MS experiments were performed in duplicate on different days to avoid day-batch effects.

Liquid chromatography-Tandem mass spectrometry

Peptides from blue native fractionation experiments were analyzed by online nanoLC-MS/MS on an Orbitrap Velos mass spectrometer coupled with an Ultimate 3000 RSLCnano System. Samples were first loaded and desalted on a nanotrap (100 μm id x 2 cm) (PepMap C18, 5 μ) at 10 μL/min with 0.1% formic acid for 10 min and then separated on an analytical column (75 μm id x 25 cm) (PepMap C18, 2μ) over a 60 min linear gradient of 5 – 42% B (B = 80% CH3CN/0.1% formic acid) at 300 nL/min, and the total cycle time was 90 min. The Orbitrap Velos was operated in standard data-dependent acquisition. The survey scans (m/z 380-1500) were acquired in the Orbitrap at a resolution of 30,000 at m/z 400, and one microscan was acquired per spectrum. The 10 most abundant multiply charged ions with a minimal intensity of 2000 counts were subject to MS/MS in the linear ion trap at an isolation width of 2 Th. Dynamic exclusion width was set at ± 10 ppm for 45 s. The automatic gain control target value was regulated at 1x106 for the Orbitrap and 5000 for the ion trap, with maximum injection time at 200 ms for Orbitrap and 100 ms for the ion trap, respectively.

Peptides from immunoprecipitation experiments were analyzed by online nanoLC-MS/MS on an Orbitrap Fusion Tribrid mass spectrometer coupled with an Ultimate 3000 RSLCnano System. Samples were first loaded and desalted on a nanotrap (100 μm id x 2 cm) (PepMap C18, 5 μ) at 10 μL/min with 0.1% formic acid for 10 min and then separated on an analytical column (75 μm id x 25 cm) (PepMap C18, 2μ) over a 120 min linear gradient of 5 – 40% B (B = 80% CH3CN/0.1% formic acid) at 300 nL/min, and the total cycle time was 150 min. The Orbitrap Fusion was operated in the Top Speed mode at 3 s per cycle. The survey scans (m/z 375-1500) were acquired in the Orbitrap at a resolution of 120,000 at m/z 200 (AGC 4x105 and maximum injection time 50 ms). The multiply charged ions (2-7) with a minimal intensity of 1x104 counts were subject to MS/MS in HCD with a collision energy at 30% and an isolation width of 1.6 Th then detected in the linear ion trap (AGC 1x104 and maximum injection time 35 ms). Dynamic exclusion width was set at ± 10 ppm for 30 s.

Analysis of mass spectrometry data

Raw data files for each blue native PAGE fractionation experiment were analyzed together using MaxQuant (version 1.5.5.1) (Cox and Mann, 2008) to identify and quantify proteins across all gel slices. Trypsin was set as digestion mode with a maximum of two missed cleavages allowed. Main search peptide tolerance was set to 20 ppm, and MS/MS match tolerance set to 0.5 Da. Carbamidomethylation of cysteine was set as a fixed modification, and acetylation at the N terminus, oxidation of methionine, and deamidation of asparagine or glutamine were set as variable modifications. Peptide and protein identifications were set at 1% FDR. Protein identification required at least one peptide for maximum coverage (Table S1). Unique and razor peptides were used for quantification. Database searches were conducted against protein sequence databases from GeneDB (Logan-Klumpler et al., 2012) (*P. falciparum* – 5431 sequences, *P. knowlesi* – 5477 sequences, *P. berghei* – 5019 sequences) for each of *P. berghei*, *P. falciparum* and *P. knowlesi*, and additionally with mouse protein sequences from UniProt (2017) (*Mus musculus* - 16950 sequences) for samples without saponin treatment. Proteins identified as potential contaminants, reverse hits or mouse proteins were removed for further analysis. The fraction corresponding to the top of the gel was removed before further analysis because of the potential to contain proteins that failed to enter the gel. Intensity scores for a protein in each fraction were normalized by dividing by the sum of protein intensities across all fractions.

Raw data files from HA immunoprecipitation experiments were analyzed using Mascot (version 2.4). Database search parameters were as above save the following: oxidation of methionine and acetylation at N terminus set as variable modifications, peptide tolerance set to 10 ppm, peptide identification set at 1% using Mascot Percolator. SAINTexpress was used to discriminate specific interactions from background binding (Teo et al., 2014). Proteins with SAINT probability score > 0.8 (FDR < 5%) were deemed specific interactors.

QUANTIFICATION AND STATISTICAL ANALYSIS

Bioinformatics Analysis

Bioinformatics analysis was performed mostly using R or Perseus (Tyanova et al., 2016).

To determine whether a protein was running above its expected molecular weight in BN-PAGE a regression line was fit to the native protein markers and their corresponding gel slices. For each quantified protein, its expected Mw was compared against the regression-predicted Mw for the fraction of greatest intensity. A protein was determined to be running above its monomeric Mw if its regression-fitted MW was more than 30% higher than its expected Mw.

Pairwise PPI scores were calculated by taking Pearson correlation scores for each pair of protein profiles in each of the 18 fractionation datasets. Reproducibility between fractionation experiments was assessed by observing the frequency of binned absolute differences between pairwise scores of two datasets.

Hierarchical clustering of protein intensity profiles was performed with Perseus (Tyanova et al., 2016). Profiles of normalized intensities for each protein across all gel slices were assembled into a dendrogram using Pearson correlation as the distance measure with complete linkage and pre-processing with k-means. Data was visualized in a heatmap.

Scoring of protein interactions by machine learning

A gold standard set of PPIs was generated using interactions taken from STRING for each of *P. berghei*, *knowlesi*, and *falciparum*. We used only protein interactions derived from experimental evidence (active interaction source "Experiments" only) with a minimum required interaction score of 0.400, and the gene IDs were mapped into the *P. falciparum* ortholog. Interactions from the three species were pooled and duplicate interactions removed, producing a set of 21,671 positive gold standard pairwise interactions. Gold standard negative interactions were produced by taking proteins from each species with the GO component term 'membrane ' or 'nucleus' into separate sets. From the 'membrane' set, proteins with descriptions or GO terms containing the patterns 'RNA', 'DNA', 'nuclear', 'nucleolar', 'chromatin', 'nucleotide', 'nuclease', 'replication', 'transcription', 'nucleic', 'helicase', 'spliceosom*', 'chromosom*', 'spindle pole', 'nucleocytoplasmic' and 'centrosom*' were removed. From the 'nucleus' set, proteins with putative transmembrane regions were removed, along with proteins containing the terms 'membrane', 'extracellular' and 'cytoplasm' in their annotation. Negative Interactions (112,032) were generated as a combination of the proteins between these sets. 205 interactions overlapped between the positive and negative sets, and were thus removed from the negative set.

The Random Forest classifier was used to elucidate high confidence PPIs from multiple data sources using the randomForest R package (Liaw and Wiener, 2001). We calculated Pearson correlation scores for each protein pair in each of the 18 fractionation datasets, and averaged them between biological replicates for each species and detergent concentration to provide 9 biochemical fractionation features, one for each experimental condition. Protein pairs with a correlation score of 0.4 or above were used in the classifier. The number of times a protein pair was detected with this criteria across all datasets was included as a feature for the classifier. The number of times a pair of proteins showed maximum intensity in the same gel fraction was also included as a feature. Additional features included domain interactions from DOMINE (Raghavachari et al., 2008; Yellaboina et al., 2011), gene co-expression (Bozdech et al., 2003; Hu et al., 2010; Modrzynska et al., 2017), co-evolution scores (Juan et al., 2008; Ochoa et al., 2015) and growth phenotype data from PlasmoGEM (Bushell et al., 2017). Only feature information pertaining to protein pairs already present in the fractionation datasets was included. Features were formatted to a binary or continuous score. For DOMINE, known domain interactions were assigned a score of 1, and high, medium and low confidence interactions from computational sources were assigned scores of 0.75, 0.5 and 0.25 respectively. To map these domain scores onto proteins, the highest score associated with domain interactions between two proteins was used. Gene co-expression scores were calculated by Pearson correlation of transcriptomic profiles from drug-induced growth perturbations (Hu et al., 2010), AP2 knockouts (Modrzynska et al., 2017) and gene expression across the asexual blood stages (Bozdech et al., 2003)in *Plasmodium* species. Co-evolution scores were taken as the output of pMirror-Tree (pMT) (Ochoa et al., 2015) and Context Mirror methodologies (Juan et al., 2008), using PPIs with p values less than 10e-5 for pMT and PPIs from Context Mirror level 10 with p values less than 10e-6. Growth phenotypes (Bushell et al., 2017) were converted to a binary score, where pairs of proteins with the same phenotype ontology were placed in one category and those with differing ontology into another. Protein pairs found common to both the resultant dataset and the gold standard were divided evenly into training and test datasets. The random forest classifier was run against the training dataset with 500 trees and allowed to choose from 4 random features at each branch split, and its performance assessed by ROC curve analysis on the test dataset. The contribution of each feature to the classifier was assessed by the mean decrease in Gini score across all trees. Protein pairs with a probabilistic output score of 0.9 or above were considered to represent putative interactions. Additional filtering criteria or raising the probabilistic score cut-off resulted in loss of experimentally-validated interactions from the network.

Receiver Operating Characteristic (ROC) curves using the gold standard were used to assess the quality of PPI datasets. To assess the validity of derived clusters, a cluster was considered to have captured an annotated protein complex from STRING or CORUM if at least half of its proteins had interactions with proteins from the same cluster that were present in these datasets. REACTOME, BioGrid and STRING pathway/interaction databases were used to assess the quality of derived interactions. Interactions were taken from these databases were treated as undirected, and mapped into *P. falciparum* gene IDs where possible. The extent to which derived interactions overlapped with these datasets was then observed.

Protein interactions were visualized as networks using Cytoscape version 3.4.0 (Shannon et al., 2003). ClusterONE was used to derive clusters from networks based on cohesiveness (Nepusz et al., 2012). Settings were chosen such as to capture the greatest number of known complexes.

A conserved *Plasmodium* PPI network was derived from the high confidence probabilistic PPI network by including edges with comigration scores present in at least one fractionation dataset from each of the three species.

Computed Gene Ontologies for process, function and component were retrieved from PlasmoDB (Aurrecoechea et al., 2009) for each species. GO term enrichment analysis for whole BN-PAGE datasets was performed with R using a Fisher's exact test with Bonferroni multiple testing correction against each *Plasmodium* species annotated genome as reference set. GO term enrichment analysis for clusters used the set of proteins seen in all clusters for a given network as reference. Clusters were deemed to recapitulate

STRING or CORUM complexes if more than 50% of cluster members were subunits of a given complex. Enrichments were also calculated for asexual blood stage parasite growth phenotype using data from PlasmoGEM (Bushell et al., 2017).

Calculation of relative evolutionary rates and gene co-evolution

Co-evolution between pairs of proteins was determined using the pMirror Tree (pMT) (Ochoa et al., 2015) and ContextMirror (Juan et al., 2008) methodologies. 3786 OrthoMCL protein families containing at least the species *P. falciparum*, *berghei*, *yoelii*, *knowlesi*, *chabaudi* and *vivax* were taken and protein FASTA sequences for all homologs retrieved. For each protein family, multiple sequence alignments were made using MUSCLE (Edgar, 2004a, b), and distance matrices produced with Protdist from PHYLIP, using the Probability Matrix from Blocks model for amino acid substitution (Veerassamy et al., 2003). For protein-protein co-evolution scores, distances matrices for each protein family were fed to pMT with the following settings: 40 groups, 0.05% chance of branch switching and 1000 branch switching iterations. As an alternate measure, distance matrices were fed to ContextMirror and level 10 protein pairs with p values less than 10e-6 were determined to be co-evolved. To assign relative evolutionary rates, an average distance was calculated for each species to *P. falciparum* across all distance matrices. For each protein family, the average distance for all species to *P. falciparum* was calculated, and divided by the average of the average distances for the same subset of species pairs. Relative evolutionary rates were used to compare protein sub-populations within interaction datasets by comparing their distributions using a Wilcoxon-Mann-Whitney test.

DATA AND CODE AVAILABILITY

All raw mass spectrometry proteomics data have been deposited to the ProteomeXchange Consortium via the PRIDE (Vizcaíno et al., 2016) partner repository (https://www.ebi.ac.uk/pride/archive/) with the dataset identifiers PXD009039 and PXD010117.

ADDITIONAL RESOURCES

Searchable database allowing easy access to the full interaction dataset: https://plasmogem.shinyapps.io/schizont_interactions/

Cell Reports, Volume *28*

Supplemental Information

Landscape of the *Plasmodium* Interactome Reveals Both Conserved and Species-Specific Functionality

Charles Hillier, Mercedes Pardo, Lu Yu, Ellen Bushell, Theo Sanderson, Tom Metcalf, Colin Herd, Burcu Anar, Julian C. Rayner, Oliver Billker, and Jyoti S. Choudhary

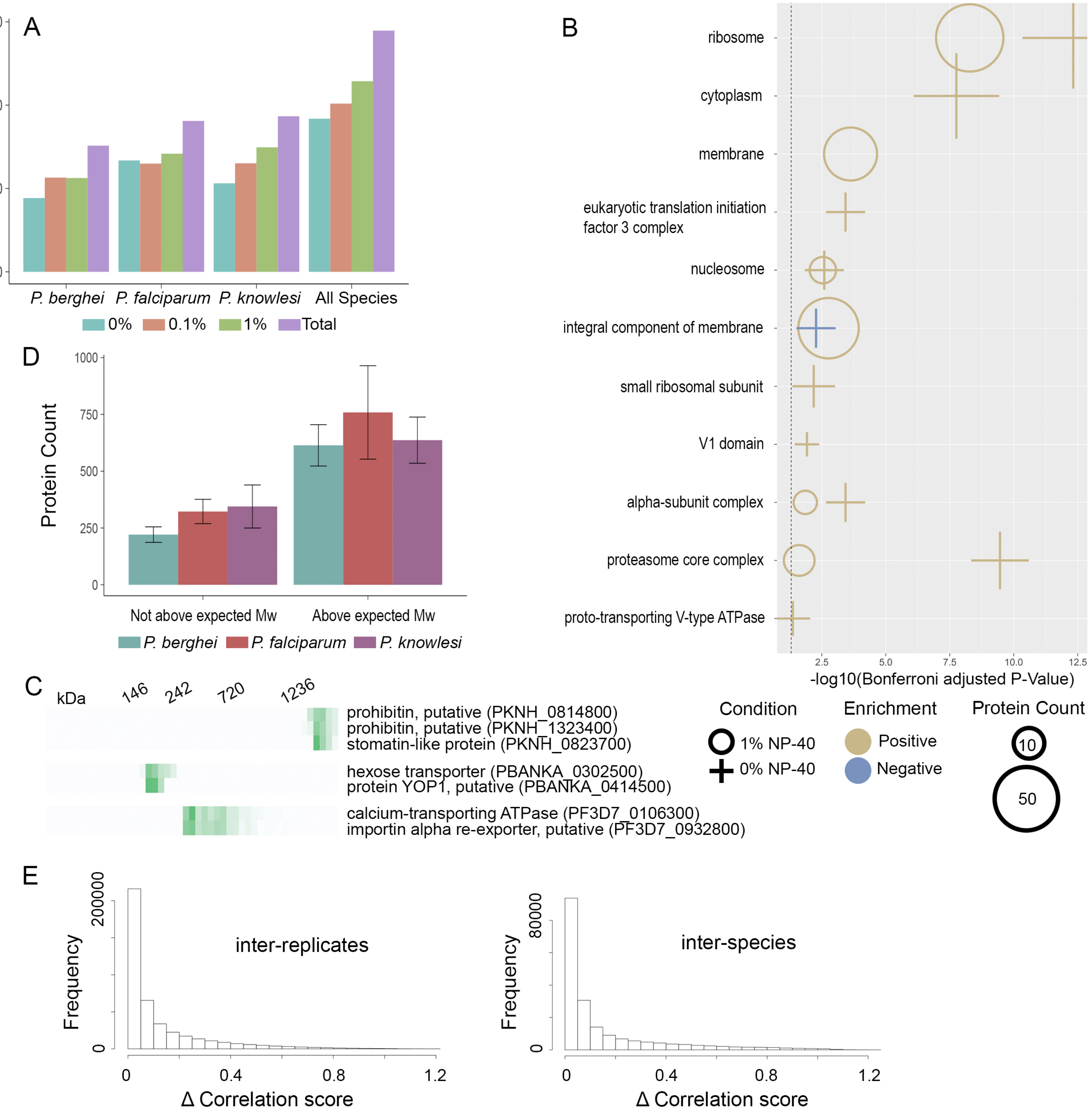

Fig. S1. Related to Figure 1. Description of the BN-PAGE fractionation data. (A) Number of proteins detected for each species and experimental condition. Bars are coloured by percentage of detergent in the lysis buffer. Protein counts for replicates are shown as union. (B) Comparison of positive and negative GO_CC term enrichments between *P. knowlesi* fractionation experiments in 0% and 1% NP-40 lysis conditions. Significance was assessed using Fisher's exact test with Bonferroni correction. The top 10 GO terms for each condition and enrichment with $p < 0.05$ are shown. The dashed line represents a p value of 0.05. (C) Migration profiles of membrane protein complexes only identified from lysates containing NP-40. (D) Number of detected proteins running above their expected BN-PAGE migration distance. Proteins with a predicted Mw at 30% higher than their literature Mw based on a regression model of known markers are considered to be running above their expected Mw. Error bars represent one standard deviation. (E) Histograms of absolute pairwise distances between co-migration scores of common pairs from replicate lysis conditions (*P. falciparum* 0.1% NP-40) and between species datasets (*P. berghei* 1% NP-40 and *P. falciparum* 0% NP-40).

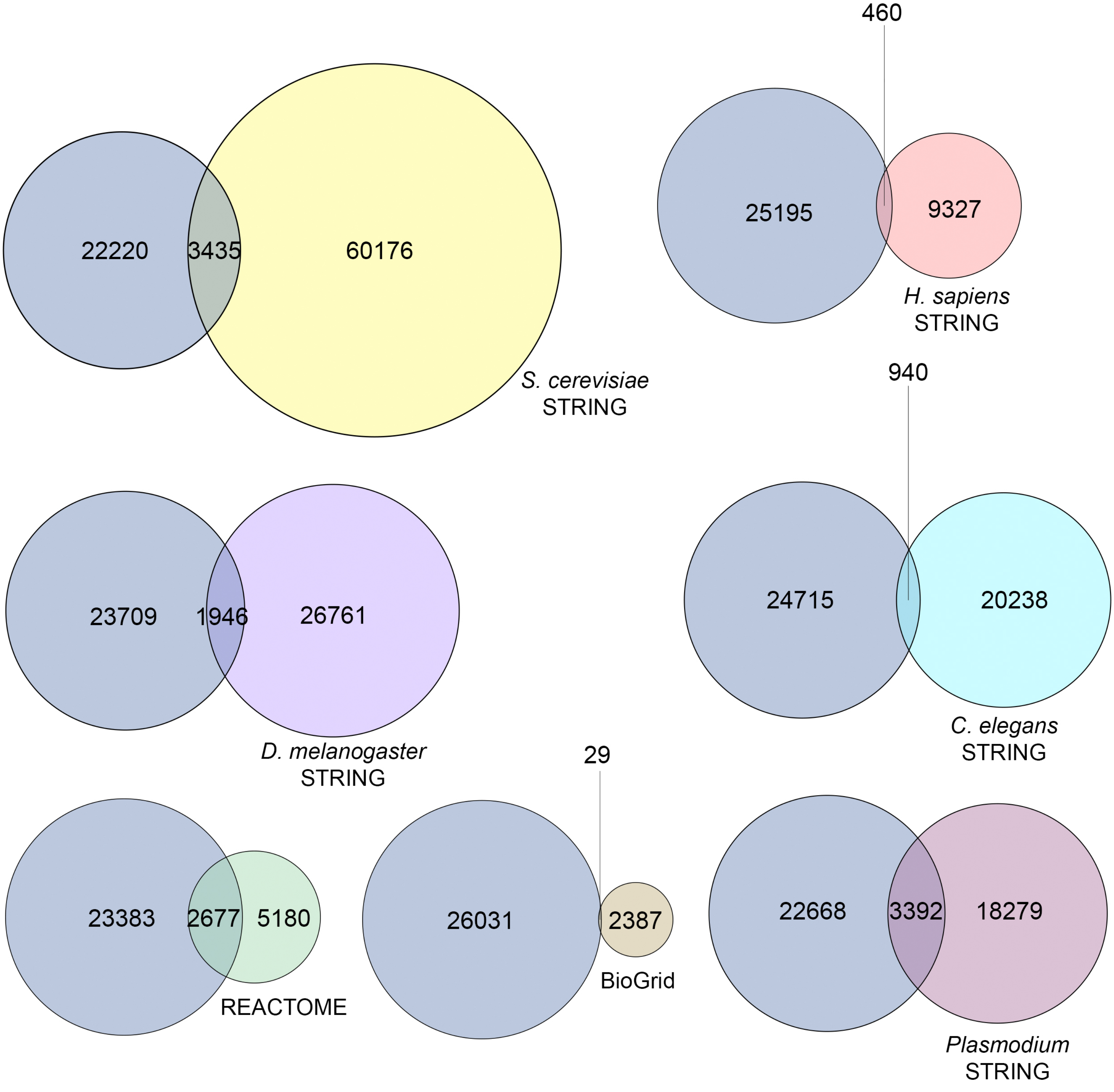

Fig. S2. Related to Figure 2. Evaluation of the high-confidence protein interaction network. Overlap between protein interactions from the random forest classifier output (grey) and PPIs annotated in public databases.
